# Coral thermal stress and bleaching enrich and restructure reef microbial communities via altered organic matter exudation

**DOI:** 10.1038/s42003-023-05730-0

**Published:** 2024-02-13

**Authors:** Wesley J. Sparagon, Milou G. I. Arts, Zachary A. Quinlan, Linda Wegley Kelly, Irina Koester, Jacqueline Comstock, Jessica A. Bullington, Craig A. Carlson, Pieter C. Dorrestein, Lihini I. Aluwihare, Andreas F. Haas, Craig E. Nelson

**Affiliations:** 1 Daniel K. Inouye Center for Microbial Oceanography: Research and Education, Department of Oceanography and Sea Grant College Program, School of Ocean and Earth Science and Technology, University of Hawaiʻi at Mānoa, Honolulu, HI 96822 USA; 2 Royal Netherlands Institute for Sea Research, Department of Marine Microbiology and Biogeochemistry, Texel, The Netherlands; 3 Scripps Institution of Oceanography, University of California San Diego, La Jolla, USA; 4 San Diego State University, San Diego, USA; 5 Department of Ecology, Evolution and Marine Biology, The Marine Science Institute, University of California Santa Barbara, Santa Barbara, USA; 6 University of California San Diego, San Diego, USA

**Keywords:** Water microbiology, Microbial ecology, Coral reefs, Microbial biooceanography

## Abstract

Coral bleaching is a well-documented and increasingly widespread phenomenon in reefs across the globe, yet there has been relatively little research on the implications for reef water column microbiology and biogeochemistry. A mesocosm heating experiment and bottle incubation compared how unbleached and bleached corals alter dissolved organic matter (DOM) exudation in response to thermal stress and subsequent effects on microbial growth and community structure in the water column. Thermal stress of healthy corals tripled DOM flux relative to ambient corals. DOM exudates from stressed corals (heated and/or previously bleached) were compositionally distinct from healthy corals and significantly increased growth of bacterioplankton, enriching copiotrophs and putative pathogens. Together these results demonstrate how the impacts of both short-term thermal stress and long-term bleaching may extend into the water column, with altered coral DOM exudation driving microbial feedbacks that influence how coral reefs respond to and recover from mass bleaching events.

## Abstract (web summary)

A mesocosm heating experiment reveals that corals stressed by bleaching and/or heating exude compositionally distinct dissolved organic matter, which in turn yields elevated microbial loads and may increase abundance of copiotrophic and putatively pathogenic bacterioplankton on stressed coral reefs.

## Introduction

Coral reef ecosystems are engineered by benthic primary producers via interactions with the water column, and unraveling these interactions is a critical step in understanding how healthy reefs function and how to prevent their degradation. Corals and algae influence the surrounding water column biogeochemistry^1–5^ by providing fixed carbon substrates to primary consumers through dissolved organic matter (DOM) exudation^6–9^. Reef benthic primary producers can release upwards of 30% of their daily photosynthate into the water column in the form of DOM, which can serve as a carbon and nutrient source for bacterioplankton^10,11^. Coral DOM exudates have unique fluorescent DOM signatures (fDOM), dissolved combined neutral sugars (DCNS) compositions, and organic compound mass spectrometry profiles compared to DOM exudates from the surrounding seawater and other benthic primary producers^12–14^. Beyond DOM serving as an energy and nutrient source for bacterioplankton, the unique quality of different DOM exudates may elicit distinct physiological changes in bacterioplankton communities as they respond to the chemical cues exuded by different benthic primary producers.

Coral DOM exudation facilitates the interaction between the coral holobiont and the surrounding bacterioplankton. Coral reef bacterioplankton exhibit chemotactic responses to a variety of DOM released by corals, including dimethylsulfoniopropionate (DMSP)^15^. DOM exudates from coral also support the growth and activity of distinct heterotrophic bacterioplankton communities^11,12,16–18^. In situ studies have identified unique metabolites and microbial communities adjacent to corals compared to the surrounding seawater^18–21^. These microbial communities are enriched in genes related to chemotaxis, motility, and signal transduction, suggesting that these regions surrounding corals contain bacterioplankton with the metabolic capacity to directly interact with corals^18–20,22^. This zone of interaction is an essential area for feedback loops between the benthos and the water column in the coral reef ecosystem, as changes in bacterioplankton may influence coral physiology^23^. Despite the burgeoning knowledge of how healthy corals influence bacterioplankton via DOM exudation, relatively little is known about if and how this relationship changes when corals are stressed.

One major stressor corals experience is ocean warming, with marine heatwaves occurring more frequently due to global climate change^24–26^. Thermal stress harms corals via bleaching, a well-documented and widespread phenomenon in which the symbiosis between corals and Symbiodinacaeae breaks down as corals are exposed to elevated temperatures for an extended period of time^27^. Mass coral bleaching has been recorded since 1979 and has increased in frequency, with a 3-fold increase in bleaching events in 2006–2012 compared to 1985-1991, restricting the time for coral reefs to recover between bleaching events^25,28,29^. Although corals can recover from bleaching, they will die if thermal stress persists^30^. Even at sub-lethal levels, coral bleaching alters the coral holobiont’s metabolism^31^, its chemistry^32^, and microbiota^33–38^.

Recent research suggests that coral-water column interactions are altered during periods of elevated temperatures^39–41^. Niggl et al.^40^ found that bleaching corals released elevated levels of particulate nitrogen (PN) and particulate organic carbon (POC) into the water column. In another study, heated *Stylophora pistillata* corals showed a change in total organic carbon (TOC) flux direction, transitioning from negative flux in healthy corals (uptake) to positive flux in heated/bleached corals (release)^41^. These alterations to water column chemistry likely shift the bacterioplankton community composition as specific taxa respond to metabolites released by stressed corals. In one study, the bacterial pathogen *Vibrio coralliilyticus* responded to DMSP as a chemotactic cue when it was released at elevated concentrations by heat-stressed coral^39^. Even under ambient conditions, coral mucus elicits a rapid chemokinetic and transcriptional response in *Vibrio coralliilyticus*, further suggesting that this pathogen uses coral chemical cues to trace and target its host^42^. Given that healthy corals exude DOM into the surrounding seawater and influence subsequent bacterioplankton dynamics, we hypothesized that (1) thermal stress and bleaching can dramatically alter DOC exudation rates and DOM composition with cascading effects on bacterioplankton growth and community structure and (2) short-term thermal stress and long-term bleaching will have different effects. These different effects will help elucidate how the succession of bleaching, from onset to recovery or death, influences the succession of DOM and bacterioplankton in situ.

## Results

### Experimental design

**Fig. 1:**
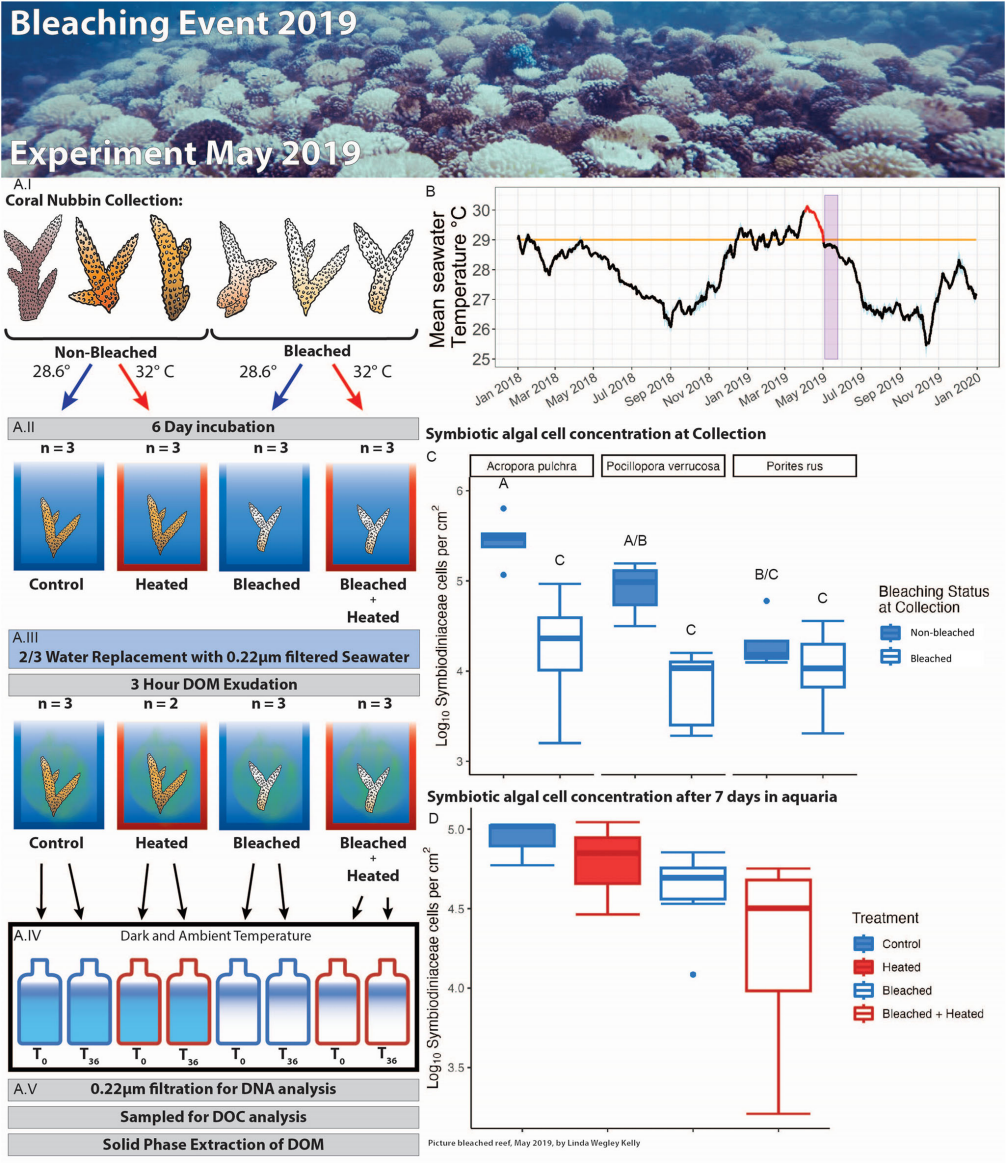
Field collections and experimental design. Non-bleached and bleached corals were collected from Moorea, French Polynesia immediately following a bleaching event (representative photo at top of forereef May 2019). **A** Overview of the experimental design (parallel ambient and heated water negative controls without corals are not shown). **A**.I Coral nubbin collection of non-bleached and bleached corals. **A**.II Six day pretreatment in ambient or heated flow-through aquaria. **A**.III 3 hour DOM exudation. **A**.IV 36 hour dark bottle incubation. **A**.V Sampling of DNA (16S), DOC, and SPE-DOM. **B** Thermal context of this experiment. Mean (black) and standard deviation (blue) seawater temperatures throughout 2018 and 2019 from three fore reef LTER sites are annotated as follows: orange line is the thermal stress accumulation threshold level of 29 °C, red line is the start of bleaching observations in April 2019, and purple block spans this experiment, which started immediately after temperatures dipped below the thermal stress accumulation threshold. **C** Starting symbiont cell concentrations of a subset of collected nubbins from the three coral species (Acropora pulchra, Pocillopora verrucosa, Porites rus) sacrificed after the three day acclimatization period prior to experiment initiation. **D** Ending mean symbiont cell concentrations in each aquaria after seven days. Coral images hand drawn by Milou Arts.

We tested these hypotheses during a mass bleaching event in Mo’orea, French Polynesia. In April 2019, the reefs of Mo’orea bleached after a prolonged period of high water temperatures^43,44^ (Fig. 1). In May 2019, immediately following this thermal stress event, we leveraged the natural distributions of recently bleached and unbleached corals to elucidate the independent and combined impacts of experimentally-induced thermal stress and recent bleaching on coral DOM exudation and subsequent bacterioplankton remineralization and growth. In brief, coral nubbins from three different species (*Pocillopora verrucosa*, *Acropora pulchra*, and *Porites rus)* assigned to both bleached and unbleached phenotypes were collected and exposed to six days of either ambient (28.6 °C) or elevated water temperatures (32 °C ± 0.2 °C) and ambient light levels in flow-through aquaria (*n* = 3 per treatment) with unfiltered water sourced from a depth of 6 m directly adjacent to the Gump Station fringing reef (Fig. 1A.I). Rather than test for species-specific differences in DOM release and bacterioplankton response, we opted to combine the 3 coral species in individual aquaria to mimic the natural composition of coral communities on Mo’orea^45^ and assess the general coral community response to thermal stress and bleaching. The combination of bleaching level and temperature yielded four treatments representing a factorial cross of prior bleaching phenotype and temperature: “Control”, “Heated”, “Bleached”, and “Bleached + Heated” (Fig. 1A.II). Additionally, two water-only control aquaria, one for each temperature treatment, were included (“Negative Control” and “Negative Control + Heated”). Because we hypothesized that recent bleaching and subsequent experimental heat stress are additive rather than interactive effects inducing four distinct coral physiologies, we considered these treatments as distinct categories in all downstream statistics. After seven days of pretreatment, the water was changed to filtered (0.22 μm) seawater, and corals were left to exude DOM for 3 hours. Coral community DOM exudates from each of the aquaria were collected (Fig. 1.A.III) and used as growth media for dark incubation dilution cultures (Fig. 1A.IV). Unfiltered back-reef seawater was used as an inoculum representative of ambient back-reef bacterioplankton communities^12^. Dilution cultures were conducted in 1 L 10% acid washed, triple milliQ rinsed (hereafter termed “acid washed”) polycarbonate bottles in the dark at ambient temperatures for 36 hours and sampled at the beginning (*n* = 3 per treatment) and end (*n* = 3 per treatment) of the incubation. We used a combination of bulk DOC measurements, flow cytometry, 16 S amplicon sequencing, and untargeted metabolomics to assess differences in the composition of DOM exudates and how these exudates altered microbial growth and community structure (Fig. 1A.V).

### Symbiodiniaceae densities

Symbiodiniaceae cell densities were used to assess individual and aquaria-wide bleaching levels of corals collected in the field and after the seven-day pretreatment, respectively. At collection, bleached corals had significantly lower Symbiodiniaceae cell densities (two-way ANOVA, *F* = 45.552, *p* = 2.67e-08). Coral species also had a significant effect on Symbiodiniaceae densities (two-way ANOVA, *F* = 4.738, *p* = 0.0137), as well as the interaction between coral species and bleaching (two-way ANOVA, *F* = 4.287, *p* = 0.0199) (Fig. 1C).

After seven days of incubation at ambient and elevated temperatures, the average Symbiodinaceae densities showed that the four coral treatments had varying degrees of bleaching (Fig. 1D). Bleached corals had significantly lower Symbiodinaceae densities (two-way ANOVA, *F* = 6.584, *p* = 0.0333). Heating did not have a significant effect on Symbiodiniaceae cell densities (*F* = 0.001, *p* = 0.9727), and neither did the interaction between heating and bleaching (two-way ANOVA, *F* = 1.284, *p* = 0.2901). Therefore, the Control treatment had the highest Symbiodiniaceae densities during the exudation experiment. Heated aquaria exhibited slightly lower Symbiodiniaceae cell densities consistent with some paling, yet still had higher cell densities than their Bleached and Bleached + Heated counterparts.

### Dissolved organic carbon

**Fig. 2:**
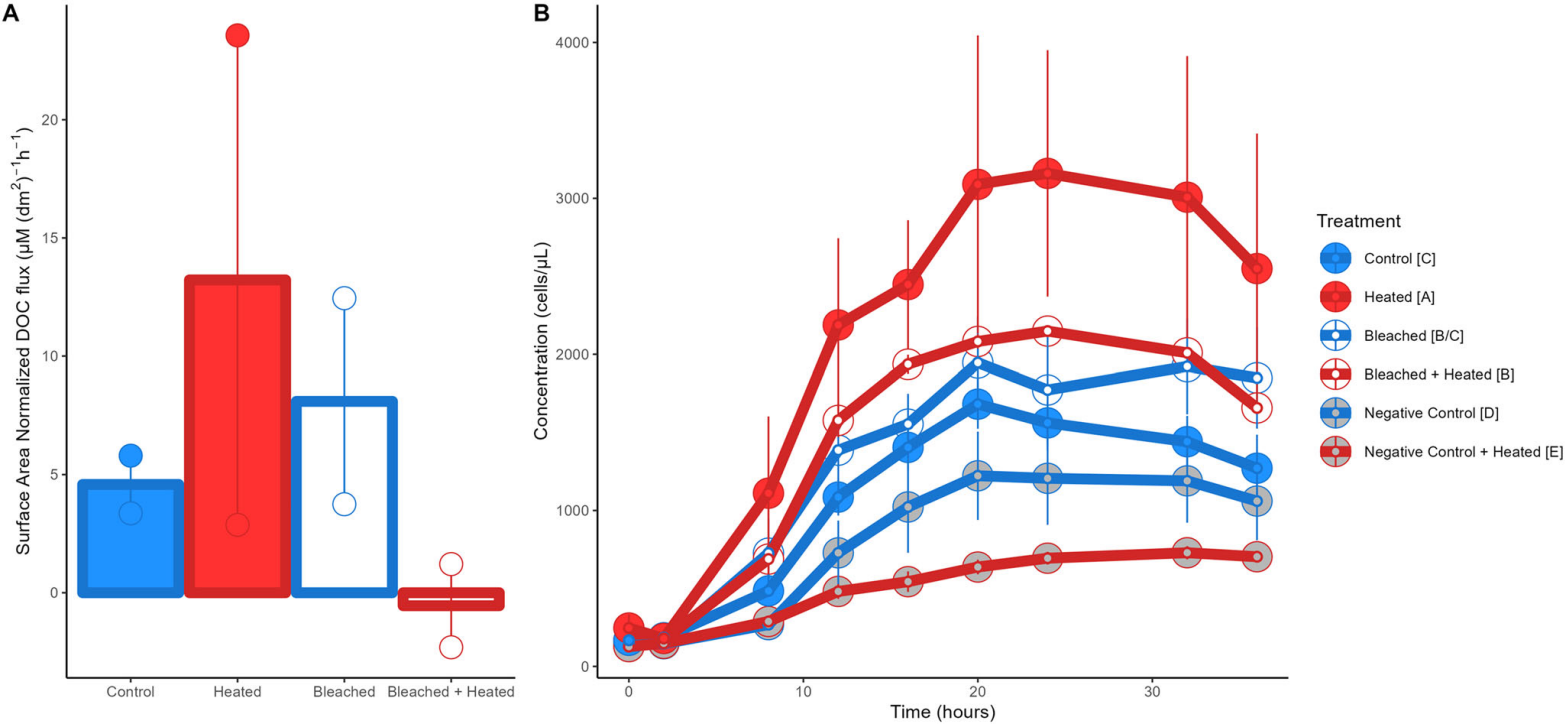
DOC exuded and microbial growth on exudates. **A** Bar plots of the mean surface area normalized DOC concentrations for the four coral treatments. Error bars indicate the standard error of the mean. **B** Bacterial growth curves for the six treatments in the 36 hour bottle incubation, error bars indicate standard error of the mean. Significant differences between treatments (Tukey post-hoc test, *p* < 0.05) are denoted by the square brackets after each treatment name in the legend.

Control corals exuded ~5 µM C (dm^2^)^−^ h^−1^ while Heated corals and Bleached corals exhibited ~289% (mean 13.22 µM C (dm^2^)^−1^ h^−1^) and 146% (mean 11.27 µM (dm^2^)^−1^ h^−1^) higher DOC fluxes. Bleached + Heated corals exhibited undetectable DOC flux. Although DOC exudation appeared to be affected by treatment, we were unable to elucidate significant differences in areal DOC flux rates possibly due to small sample size and short exudation times (Kruskal–Wallis chi-squared = 4.1667, *p* = 0.244) (Fig. 2A). Coral treatments generally had higher raw DOC concentrations than the water controls, although this effect was not significant (Kruskal–Wallis chi-squared = 9.3187, *p* = 0.09) (Figure S2).

### Microbial growth

Flow cytometry revealed distinct microbial growth patterns between the DOM treatments. Two-way ANOVAs were used to test the effect of both time point and treatment on bacterioplankton cell concentrations. We found that bacterioplankton concentrations significantly increased through time in the dark bottle incubations (two-way ANOVA, *F* = 104.372, *p* < 2.2E-16) and differed significantly between treatments (two-way ANOVA, *F* = 53.685, *p* < 2.2E-16) (Fig. 2B). All coral treatments had significantly higher cell concentrations than both the Negative Control and the Negative Control + Heated (Tukey post-hoc test, *p* < 0.05). Within the coral DOM treatments, Heated exudates yielded significantly higher bacterioplankton concentrations than the Control (Tukey post-hoc test, *p* < 0.05), with bacterioplankton grown on Heated DOM reaching concentrations of 3,160,000 cells/mL (± 790,000), nearly double that of the Control (1,562,000 cells/mL ± 137,000) (Fig. 2B). Bacterioplankton specific growth rate also differed significantly between the treatments (one-way ANOVA, *F* = 6.363, *p* = 0.005) (Fig. S3).

### Microbial community structure

**Fig. 3:**
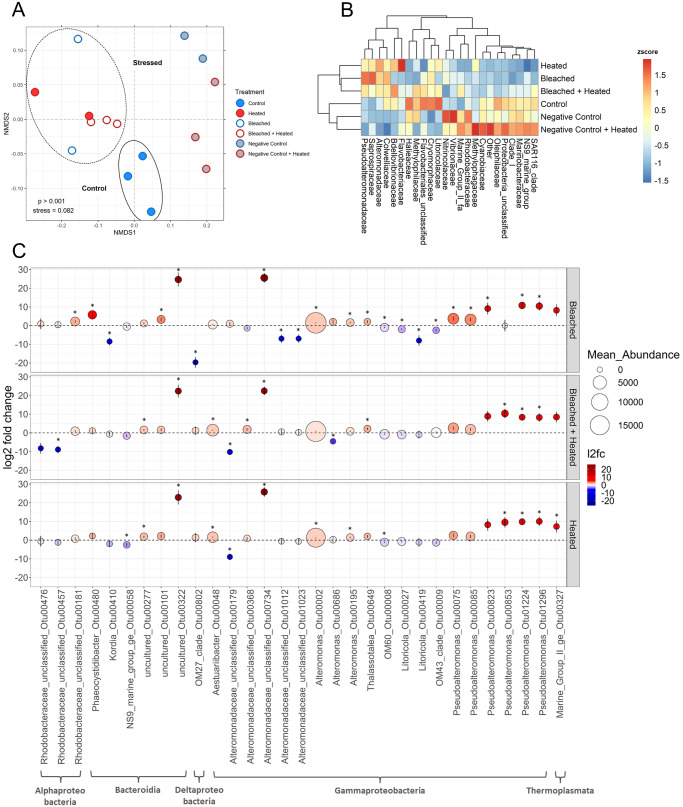
Changes in microbial communities in response to exudates. **A** Non-metric multidimensional scaling of microbial community samples using Unifrac distances derived from 16 S amplicon data. A dashed ellipse denotes the 3 coral stress treatments while a solid ellipse denotes the coral Control treatment. **B** Two-way heatmap of the most abundant bacterial families in each treatment. Abundant families were defined as: relative abundance ≥ 0.005 in samples ≥ 3 or a relative abundance ≥ 0.1 in samples ≥ 1. Each cell represents the z-scored mean relative abundance of a given family in a treatment. Cells are colored according to z-score, with warmer colors indicating enrichment and cooler colors indicating depletion. Clustering was performed using Euclidian distances. **C** Visualization of the 31 OTUs determined to be significantly differentially abudant (DA) in at least one of the three stress treatments compared to Control samples by DESeq2. Dotplot of the log2 fold-change (l2fc) values for the 31 significantly DA OTUs in the three coral stress treatments. Each dot represents a given OTU in a given treatment. Dot height on the y-axis and color correspond to log2 fold-change values. Error bars depict the standard error of each log2 fold-change value calculated by DESeq2. Dot size corresponds to mean raw abundance. Each OTU is labeled according to its class, family, and Genus_OTUNumber on the x-axis. Asterisks denote a significant DA OTU in a treatment.

The DOM derived from the six treatments yielded distinct microbial communities after 36 hours of growth. There was a significant change in microbial communities from the start to the end of the bottle incubations, indicating that communities changed through the bottle incubation and were not simply reflective of the starting communities (PERMANOVA, *F* = 72.033, *R*^2^ = 0.71, *p* ≤ 0.001, Fig. S4). After 36 h, bacterioplankton grown on coral exudates were significantly different from those grown on water negative controls (PERMANOVA F = 8.679, *R*^2^ = 0.40, *p* ≤ 0.001), and overall DOM treatment had a significant effect on microbial community structure (PERMANOVA *F* = 4.637, *R*^2^ = 0.72, *p* ≤ 0.001) (Fig. 3A). Within the coral treatments, Controls maintained distinct community structure from the Heated, Bleached, and Bleached + Heated communities, hereafter collectively referred to as “stressed” (PERMANOVA *F* = 5.501, *R*^2^ = 0.41, *p* = 0.009). There was overlap within the stressed communities and a consistent shift in ordination space away from the Controls, indicating a potentially conserved change that occurs in microbial communities in response to stressed coral exudates.

Many eutrophic coastal-associated bacterial families were overrepresented in the negative controls including Nitrincolaceae and Methylophagaceae^46^, as well as the common coral reef-associated family Rhodobacteraceae^47–51^. Coral DOM microbial communities were dominated by the families Alteromonadaceae and Rhodobacteraceae, although there was substantial variation between the four treatments (Fig. 3B, Table S1). Control corals maintained distinct microbial communities from both negative controls and stressed corals, overrepresented in the families Cryomorphaceae, Litoricolaceae, and Halieaceae. Alteramonadaceae, a common marine copiotrophic family^52^, was lowest in communities grown on Control exudates (28.3%) and substantially higher in the communities growing on the stressed coral exudates (46.6–38.6%). Pseudoalteromonadaceae also increased in abundance from Control communities to stressed communities (3.3% in Control, and a mean of 9.1% in stressed). The common coral reef-associated family Rhodobacteraceae was higher in the Control (21.3%) and lower in the three stressed treatments (13.7–19.4%). Another common coral reef-associated family, Cryomorphaceae, was abundant in the Control treatment (13.0%) and then reduced in abundance in the stressed treatments (5.6–7.2%). Microbial community alpha diversity metrics (observed sequences, Chao diversity, Shannon diversity, and Shannon’s Evenness) were assessed after 36 hours of growth (Fig. S5), with only Shannon diversity being significantly affected by treatment (one-way ANOVA, *F* = 3.914, *p* = 0.037).

### Bacterial taxa differential abundance analysis

Multivariate analysis demonstrated that stressed corals enriched distinct bacterioplankton communities compared to their Control counterparts. In order to directly elucidate which specific bacterial taxa were driving these differences, we performed DESeq2, (see supplementary methods) on a subset of the data that only included the four coral DOM treatments^53^, yielding a final count of 159 OTUs to be run through DESeq2. The log2 fold-change of OTU abundances was calculated between the three coral stress treatments and the coral controls to elucidate OTUs that were differentially abundant when corals were bleached and/or heated (Supplementary Data 1) (Fig. 3C and S6).

A total of 31 significantly differentially abundant bacterial OTUs (19.5%) were identified by DESeq2 (DESeq2, log2 fold-change > 0, *p* ≤ 0.05 after FDR adjustment, Table 1, Figs. 3C, S6, and S7). These OTUs belonged to four bacterial and one archaeal class: Alphaproteobacteria, Gammaproteobacteria, Bacteroidia, Deltaproteobacteria, and Thermoplasmata, with the majority (64.5%) of differentially abundant OTUs belonging to Gammaproteobacteria. Within Gammaproteobacteria, there was a significant enrichment of numerous abundant Alteromonadaceae and Pseudoalteromonadaceae OTUs in at least one of the three treatments (75% of all differentially abundant Gammaproteobacteria OTUs).

**Table 1:** **:** DESeq2 results for the 31 OTUs that were significantly (*p* < 0.05 after FDR) differentially abundant in at least one coral stress treatment relative to coral Controls.

OTU	Heated l2fc	Bleached l2fc	Bleached + Heated l2fc	Class	Order	Family	Genus
Otu01224	**9.847**	**10.793**	**8.362**	Gammaproteobacteria	Alteromonadales	Pseudoalteromonadaceae	*Pseudoalteromonas*
Otu01296	**10.044**	**10.459**	**8.233**	Gammaproteobacteria	Alteromonadales	Pseudoalteromonadaceae	*Pseudoalteromonas*
Otu00322	**22.866**	**24.671**	**22.365**	Bacteroidia	Chitinophagales	Saprospiraceae	unclassified
Otu00734	**25.799**	**25.542**	**22.461**	Gammaproteobacteria	Alteromonadales	Alteromonadaceae	Alteromonadaceae unclassified
Otu00195	**1.369**	**1.622**	0.846	Gammaproteobacteria	Alteromonadales	Alteromonadaceae	*Alteromonas*
Otu00002	**1.293**	**1.469**	0.776	Gammaproteobacteria	Alteromonadales	Alteromonadaceae	*Alteromonas*
Otu00008	**−1.150**	**−0.991**	**−**0.724	Gammaproteobacteria	Cellvibrionales	Halieaceae	OM60
Otu00179	**−8.978**	1.002	**−10.230**	Gammaproteobacteria	Alteromonadales	Alteromonadaceae	Alteromonadaceae unclassified
Otu00277	**1.820**	1.184	**1.551**	Bacteroidia	Flavobacteriales	Flavobacteriaceae	unclassified
Otu00048	**1.561**	0.592	**1.434**	Gammaproteobacteria	Alteromonadales	Alteromonadaceae	*Aestuariibacter*
Otu00853	**9.520**	0	**10.366**	Gammaproteobacteria	Alteromonadales	Pseudoalteromonadaceae	*Pseudoalteromonas*
Otu00058	**−2.524**	**−**0.482	**−**1.519	Bacteroidia	Flavobacteriales	NS9 marine group	NS9 marine group
Otu00823	8.240	**9.123**	**8.912**	Gammaproteobacteria	Alteromonadales	Pseudoalteromonadaceae	*Pseudoalteromonas*
Otu01012	**−**0.587	**−6.976**	0.518	Gammaproteobacteria	Alteromonadales	Alteromonadaceae	Alteromonadaceae unclassified
Otu01023	**−**0.687	**−6.964**	0.238	Gammaproteobacteria	Alteromonadales	Alteromonadaceae	Alteromonadaceae unclassified
Otu00419	**−**1.158	**−7.989**	**−**0.929	Gammaproteobacteria	Oceanospirillales	Litoricolaceae	*Litoricola*
Otu00327	7.309	8.194	**8.464**	Thermoplasmata	Marine Group II	Marine Group II	Marine Group II
Otu00476	**−**0.576	0.964	**−8.258**	Alphaproteobacteria	Rhodobacterales	Rhodobacteraceae	Rhodobacteraceae unclassified
Otu00649	1.986	**2.102**	**2.205**	Gammaproteobacteria	Alteromonadales	Colwelliaceae	*Thalassotalea*
Otu00101	2.185	**3.294**	1.634	Bacteroidia	Chitinophagales	Saprospiraceae	unclassified
Otu00181	0.778	**2.221**	0.908	Alphaproteobacteria	Rhodobacterales	Rhodobacteraceae	Rhodobacteraceae unclassified
Otu00027	**−**0.714	**−1.944**	**−**0.899	Gammaproteobacteria	Oceanospirillales	Litoricolaceae	*Litoricola*
Otu00410	**−**1.992	**−8.512**	**−**0.633	Bacteroidia	Flavobacteriales	Flavobacteriaceae	*Kordia*
Otu00480	2.223	**5.767**	1.107	Bacteroidia	Flavobacteriales	Cryomorphaceae	*Phaeocystidibacter*
Otu00075	2.553	**3.699**	2.546	Gammaproteobacteria	Alteromonadales	Pseudoalteromonadaceae	*Pseudoalteromonas*
Otu00085	1.965	**3.203**	1.797	Gammaproteobacteria	Alteromonadales	Pseudoalteromonadaceae	*Pseudoalteromonas*
Otu00802	1.328	**−19.636**	1.189	Deltaproteobacteria	Bdellovibrionales	Bdellovibrionaceae	OM27
Otu00009	**−**1.294	**−2.496**	0.163	Gammaproteobacteria	Betaproteobacteriales	Methylophilaceae	OM43
Otu00368	0.928	**−**1.382	**1.855**	Gammaproteobacteria	Alteromonadales	Alteromonadaceae	Alteromonadaceae unclassified
Otu00457	**−**1.196	0.495	**−8.927**	Alphaproteobacteria	Rhodobacterales	Rhodobacteraceae	Rhodobacteraceae unclassified
Otu00686	0.234	1.952	**−4.512**	Gammaproteobacteria	Alteromonadales	Alteromonadaceae	*Alteromonas*

Significant log2 fold change (l2fc) values are highlighted in bold.

In all three stress treatments, there was a significant enrichment of two *Pseudoalteromonas* OTUs (1224 and 1296), one *Alteromonas* OTU (734), and one unclassified Saprospiraceae OTU (322). All three stress treatments also showed an enrichment of a highly abundant *Alteromonas* OTU^2^, although this was only statistically significant in the Bleached and Heated treatments. There were 12 differentially abundant OTUs found only to be significant in the Bleached treatment including significant enrichment in members of the genus *Psuedoalteromonas* (OTUs 75 and 85), unclassified Saprospiraceae (OTU 101), and *Phaeocystidibacter* (OTU 480), and significant reductions in members of the genus *Litoricola* OTUs 27 and 419) and unclassified Alteromonadaceae (OTUs 368, 1012, and 1023). The two heated treatments had significant enrichment in OTUs of the genus *Psuedoalteromonas* (OTU 853), *Aestuariibacter* (OTU 48), and an unclassified Flavobacteriaceae (OTU 277). The two bleached treatments showed significant enrichment of OTUs in the genera *Pseudoalteromonas* (OTU 823) and *Thalassotalea* (OTU 649). Visualization of pairwise co-occurrence patterns of OTUs from the final time point reinforces these conclusions (Fig. S8). OTUs enriched in the coral stress treatments, including Alteromonadaceae, Pseudoalteromonadaceae, and Saprospiraceae clustered together in the upper portion of the network and showed a high degree of connectivity with each other (i.e., OTU 1224 and OTU 75), yet limited significant positive correlations with OTUs in the network that were enriched in coral controls and/or negative water controls.

### Metabolomes

**Fig. 4:**
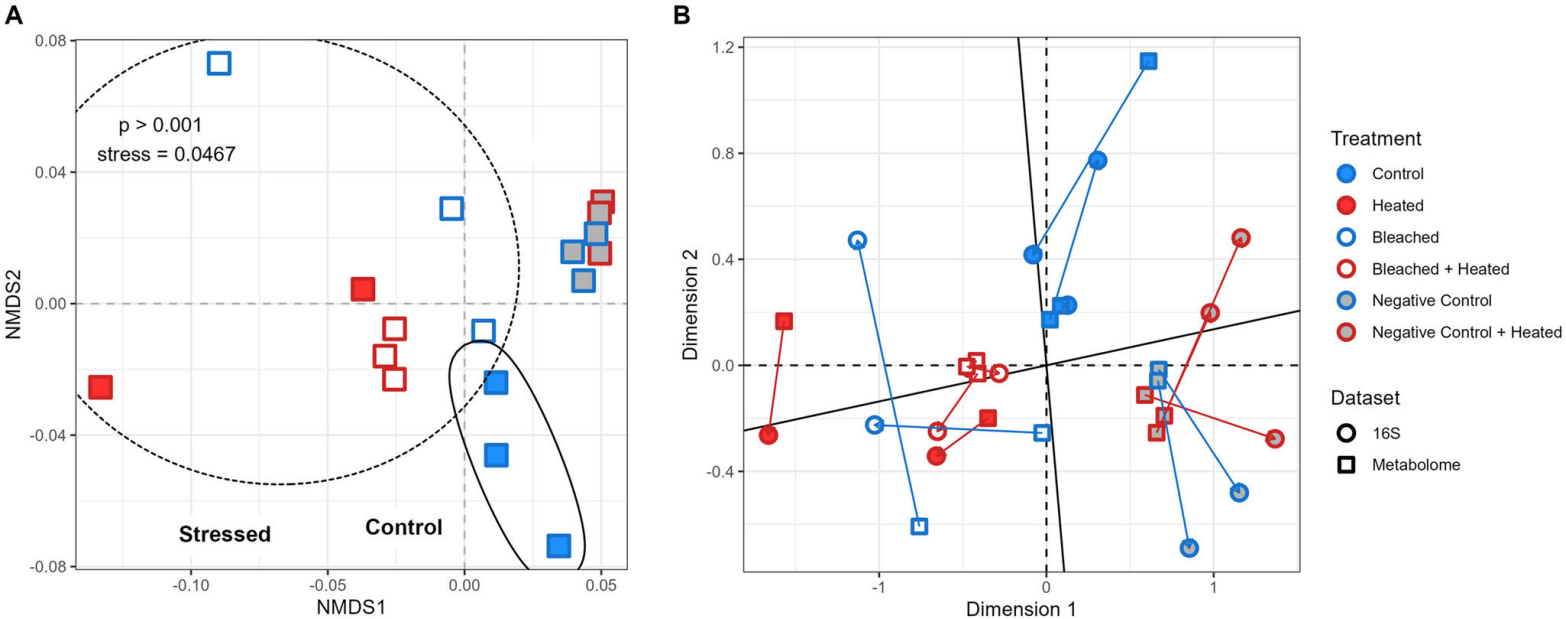
Compositional differentiation of coral exudates (as measured by untargeted tandem mass spectrometry) and correspondence with composition of microbial communities grown on exudates. **A** Non-metric multidimensional scaling plot of t0 metabolomic samples using bray-curtis dissimilarity. A dashed ellipse denotes the 3 coral stress treatments, while a solid ellipse denotes the coral Control treatment. **B** Procrustes visualization of multivariate metabolomic and microbial samples. Arrows point from metabolomic samples to corresponding microbial samples.

Another potential driver of bacterioplankton enrichment in this study, beyond DOC concentration, was compositional differences in the DOM exudates. Untargeted metabolomics was performed to assess the impact of DOM quality on microbial community structure. The exo-metabolomes consisting of the extracted ion-chromatograms (XIC values) of each metabolite were used to generate a Bray–Curtis dissimilarity matrix to test multivariate differences between the six treatments at time point *T* = 0. The exo-metabolomes indicated that different treatments produced compositionally distinct DOM exudates following the three-hour DOM exudation (PERMANOVA, *F* = 1.7847, *R*^2^ = 0.44788, *p* ≤ .001) (Fig. 4A). Consistent with the bacterioplankton data, coral samples clustered separately from the water negative controls and within the coral treatments, coral Controls maintained distinct metabolomes compared to the three stress treatments (one-way PERMANOVA *F* = 1.3799, *R*^2^ = 0.37162, *p* = 0.005). Multivariate comparisons between the 16 S rDNA and metabolomics distance matrices confirmed that DOM composition significantly correlated with bacterioplankton community structure (Procrustes correlation = 0.8612, significance = 0.001; Mantel *R* = 0.5993, significance = 0.001) (Fig. 4B), indicating that bacterioplankton community changes may be a response to shifts in DOM exudate quality as well as quantity.

## Discussion

This study presents a comprehensive assessment of how short-term thermal stress and long-term bleaching, separately and in combination, influence reef bacterioplankton via DOM exudation by coral communities. Our results indicate that thermally-induced bleaching events both increase and alter coral community DOM exudation with cascading impacts on reef bacterioplankton dynamics that potentially hamper coral resistance to and recovery from bleaching.

Heated corals had the highest DOC fluxes of roughly 15 µM C (dm^2^)^−1^ h^−1^, marking a substantial contribution to reef DOC that is between 8.4 and 13.6 times higher than previously documented coral DOC release rates in Mo’orea^11,16^. Photosynthesis reactions, which contribute to a portion of the DOC released by corals, are sensitive to increases in temperature^54,55^ and elevated temperatures are known to speed up enzymatic reaction rates. It is possible that maintenance of intact coral-algal symbiosis in the face of elevated temperatures may lead to higher rates of carbon fixation and higher DOC release^54^. This is supported by Hillyer et al., 2017^56^ who found that glucose, a major product of dinoflagellate-cnidarian symbiosis^57–59^, appears to increase in thermally stressed corals.

Both Bleached and Bleached + Heated corals had lower DOC release rates that were not significantly different from DOC release rates of coral Controls. Without their endosymbionts, corals are known to catabolize internal carbon stores, especially lipids, to meet their energetic demands that are no longer satisfied by photoautotrophy^60–65^. DOM mobilization from internal stores could yield similar DOC release compared to healthy corals, despite reduced densities of Symbiodiniaceae.

DOM exudates from Heated, Bleached, and Bleached + Heated corals appear to be labile, i.e., readily mobilized by bacterioplankton into metabolic pathways^66^. In general, DOM derived from coral treatments grew more concentrated microbial communities with faster growth rates than the Negative Controls. Heated coral DOM produced higher microbial growth rates, resulting in double the bacterioplankton concentrations compared to the coral Control treatment (3,000,000 cells/mL). This rapid growth of microbes on heated coral DOM could have negative impacts on corals in situ, where already stressed individuals are further stressed through the generation of hypoxic zones from high levels of bacterial respiration^18,23,67–70^. Despite the lower DOC release rates than Heated corals, both Bleached and Bleached + Heated DOM increased bacterioplankton concentrations compared to coral Controls. This suggests that changes in the composition DOM released by stressed corals may generally increase microbial growth, regardless of DOC concentration.

Bacterioplankton communities fed stressed coral DOM show a conserved, directional shift of microbial community structure away from bacterioplankton associated with healthy coral DOM, indicating a potential universal response of bacterioplankton to coral stress, whether that be heating, bleaching, or both. Differential abundance analysis of OTUs using DESeq2 revealed that this universal stress response in bacterioplankton communities was largely driven by an enrichment of copiotrophs and putative pathogens.

The three stressed treatments were highly enriched in bacteria commonly associated with large inputs of labile organic matter, including three *Alteromonas* OTUs (OTUs 2, 195, and 734, although OTUs 2 and 195 were only significantly enriched in Bleached and Heated), one Saprospiraceae OTU (OTU 322, significantly enriched in all three treatments), and two *Pseudoalteromonas* OTUs (OTUs 1224 and 1296, significantly enriched in all three treatments). These three taxa are common copiotrophs associated with large inputs of organic matter including from algal blooms^52,71–74^, in controlled incubations^75–77^, and in response to pulses of POM on coral reefs during coral spawning^78^. The enrichment of these OTUs in all coral stress treatments suggests a universal response of corals to heating and/or bleaching that induces the release of labile organic matter, which then rapidly enriches heterotrophic bacteria in the plankton. Through the lens of *r-* and *K-*selection, the release of surplus labile organic matter by stressed corals proliferates *r*-selected copiotrophs that rapidly outcompete the *K*- selected taxa that are often associated with marine oligotrophic, and specifically coral reef, systems.

The universal stress response in bacterioplankton communities was also driven by an enrichment of putative pathogens, specifically in the families Colwellieaceae and Pseudoalteromonadaceae. OTU 649, belonging to the genus *Thalassotalea*, was enriched in all three treatments (only significantly so in Bleached + Heated and Bleached) and shared a 100% 16 S rDNA sequence identity with a *Thalassomonas* bacteria that induced severe bleaching in corals after only 24 hours^79^. *Pseudoalteromonas* OTU 823 was also highly enriched in all three stressed coral treatments (again only significantly so in Bleached + Heated and Heated) and was closely related (100% 16 S rRNA identity) to *Pseudoalteromonas piratica*, which has been identified as the causative agent of the coral disease “acute *Montipora* White Syndrome”^80^. Enrichment of putative pathogens in the stress treatments could be driven by a positive association with coral stress metabolites^39^ or because stressed corals lack the production of defense molecules; in either case, the enrichment of these pathogenic taxa could be detrimental to coral health.

Although we are not aware of any studies that have examined how stressed coral DOM alters bacterioplankton in bottle incubations, Sun et al.^81^ examined the impact of coral bleaching on bacterioplankton in a flow-through aquaria setting and corroborate many of the observations found here: copiotrophic taxa (in this case Flavobacteriaceae) increased in heated coral treatments and there is an uptick in pathogenic gene functions in bacterioplankton after seven days of heating.

One potential driver of microbial changes in this study beyond bulk DOC differences is qualitative differences in the DOM exudates. The composition of DOM has been shown to shape microbial communities in numerous systems including coral reefs^12^, the open ocean^82^, and synthetic microbial consortia^83^. The same patterns hold in this study; different DOM treatments yielded different microbial communities, with DOM metabolomic composition significantly correlated with microbial community structure. This suggests that changes in the quality of coral DOM exudates, not just quantity, shapes bacterioplankton communities during thermally-induced bleaching.

The four coral treatments clustered away from the two negative water controls, aligning with previous observations that corals alter water column DOM composition^12–14^. Importantly, there was no distinction between the ambient and heated negative controls, suggesting that temperature alters DOM quality indirectly via coral exudation rather than by directly acting on the water column. Within the coral treatments, the three stressed treatments clustered away from the Control corals, suggesting that stressed corals altered the quality of their DOM exudates. The consistent clustering of stressed coral treatments away from healthy coral treatments in both the microbial and metabolomics data hints at the potential release of universal stress metabolites by corals experiencing a variety of heating/bleaching regimes, leading to conserved shifts in the DOM pool and in turn conserved shifts in bacterioplankton communities. These metabolites would be present regardless of the specific stress regime and, once exuded into the water column, would fuel the consistent growth of opportunistic families and putative pathogens. Further studies should aim to directly assess this possibility.

In this study, we used a mixture of three common Mo’orea coral species in each aquaria in order to investigate the coral community’s DOM exudation response to thermal stress/bleaching. Different coral species can exude different DOM quantities and compositions and yield slightly different microbial communities, although differences between coral species are smaller than differences between broader benthic “guilds”^12,14^. The current experiment did not allow us to investigate the species-specific response to heating and bleaching. While we hypothesize that increased and altered DOM exudation is a universal response to heating and bleaching by coral communities, future studies should investigate species-specific differences. If there are substantial differences in DOM exudation and subsequent bacterioplankton growth between coral species, then the composition of the reef benthos might influence the reef-wide ecological impact and response to thermal and bleaching stress.

Our data suggest a positive feedback mechanism in which thermally stressed and/or bleached corals release DOM that enriches high abundances of rapidly growing copiotrophs and putative pathogens, which can then potentially harm the coral via hypoxia due to microbial respiration or through coral disease. A similar mechanism has been observed on algae-dominated reefs; high algal benthic cover quantitatively increases and qualitatively changes DOM release, which in turn fosters a more copiotrophic microbial community with higher microbial biomass and energy use^47^. This process, termed “microbialization”, is part of the broader DDAM (DOM, Disease, Algae, and Microbes) negative feedback loop in which microbialization harms coral through disease (pathogens) and hypoxia (copiotrophs), further promoting algal dominance on the reef^47,67,84^. In much the same mechanism as the DDAM model, corals may negatively impact their own resistance to/recovery from thermally-induced bleaching via DOM exudation and subsequent bacterioplankton enrichment.

This study used a sealed, controlled bottle system to accurately measure DOC and microbial growth characteristics. However, in situ conditions are vastly different from bottles or flow-through mesocosms; physical dynamics like reef depth, water flow, and residence time, as well as the relative abundance of specific coral species on a reef, all likely impact the degree to which our observed findings translate to in situ impacts.

This study did not take into account increased temperatures during microbial growth, only the impacts of DOM. Elevated temperatures could further amplify this feedback loop by increasing microbial metabolic rates, which could be an additional factor for a rapid switch to copiotrophic communities and higher microbial abundances that were not observed at ambient incubations. The combination of elevated temperatures and increased DOM could also rapidly accelerate microbial respiration, resulting in more severe hypoxia than under ambient temperatures.

**Fig. 5:**
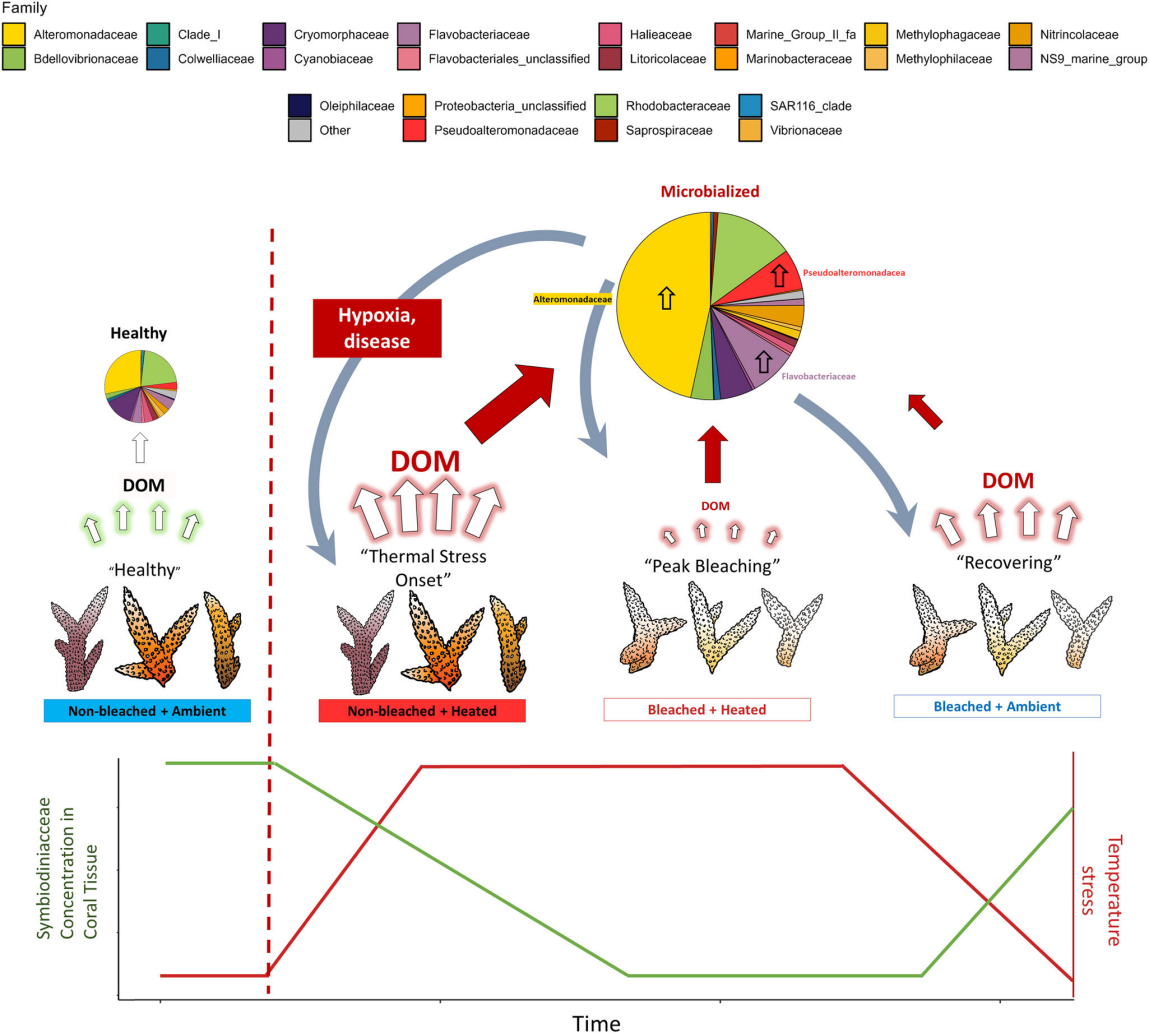
Conceptual representation of biogeochemical changes during a coral bleaching event. Bleaching progresses from left to right, with SST values increasing until their peak and then returning to ambient values. Corals experience a change in physiological state and symbiont densities through the thermal anomaly, going from “Healthy” to “Thermal Stress Onset” to “Peak Bleaching” and lastly, “Recovering”. The associated treatment names from our experiment are written below the corals. Symbiont densities for each treatment are plotted with temperature, with densities decreasing through “Peak Bleaching” and then increasing slightly in “Recovering.” DOM flux is highest at “Thermal Stress Onset”, indicated by the size of the arrows pointing from corals to “DOM”. In all 3 of the stressed treatments, bacterioplankton communities shift towards a “microbialized” state marked by increased cell counts (indicated by the size of the arrow pointing towards the pie chart) and a greater relative abundance of copiotrophs and pathogens, namely in the Alteromonadaceae, Pseudoalteromonadaceae, and Flavobacteriaceae families. We propose that these microbialized communities derived from stressed coral DOM exudates further harm the corals via hypoxia from increased bacterial loads and disease from the uptick in bacterial pathogens. The state of microbialization is most pronounced at the onset of thermal stress, which may push corals towards more severe bleaching and ultimately, mortality. Coral images were hand-drawn by Milou Arts and digitized for use in the manuscript.

The ecological implications of this study can be understood by situating the four coral treatments within the context of an in situ reef experiencing elevated water temperatures (Fig. 5). The four experimental treatments can represent four phases of thermally-induced bleaching on a coral reef, from ambient (Control) to thermally stressed (Heated) to actively bleaching (Bleached + Heated) to recovering (Bleached). In all three of the stressed coral DOM treatments, there was a marked change in DOM exudation that drove an enrichment of copiotrophs and putative pathogens in the bacterioplankton. In the above ecological interpretation of the treatments, this indicates that the aforementioned positive feedback mechanism will be present throughout various stages of a thermal anomaly, hampering both coral resistance to and recovery from bleaching via disease and hypoxia at both the onset and termination of marine heatwaves.

Importantly, the highest concentration of DOC exudate and greatest growth of bacterioplankton was in the Heated treatment. This indicates the feedback mechanism is most pronounced at the onset of coral thermal stress. Water column pathogens and copiotrophs, sniffing out and gorging on thermally stressed coral DOM exudate, may push individual corals towards more severe bleaching. If a single coral bleaches, this mechanism may have minimal impact on the water column biogeochemistry, but if an entire reef experiences elevated temperatures, the large flux of labile DOM into the water column could propagate a reef-wide shift in microbial communities that may prevent coral recovery. Additionally, coral mortality as a result of bleaching and the aforementioned mechanisms could lead to a further pulse of organic matter fuel into the water column, exacerbating the already adverse situation and turning bleaching reefs into a dead zone.

Assessing the effect of thermally-induced coral bleaching on water column dynamics reveals that stressed corals enrich a glut of copiotrophic, putatively pathogenic bacteria in the plankton via DOM exudation. Based on these results we hypothesize that at the coral colony level these effects may reduce a corals’ ability to resist and recover from thermally-induced bleaching. When our results are translated to a reef-wide scale, we predict that thermal anomalies and mass bleaching events could sharply alter reef water biogeochemistry, carbon flux, microbial communities, and ecosystem health. In this dramatic positive feedback loop, DOM is the herald of the change, translating shifts in coral physiology to shifts in water column dynamics. At the moment, this dynamic remains unrecognized and the effects understudied. To fully understand how complex coral reef ecosystems respond to marine heatwaves, producer-DOM and microbe-DOM dynamics must be taken into account.

## Methods

### Experimental design

#### Field collection

Coral nubbins from three different species (*Pocillopora verrucosa*, *Acropora pulchra*, and *Porites rus)* were collected in Mo’orea, French Polynesia on May 8th, 2019 immediately following a bleaching event (Fig. 1B). For more on this bleaching event and temperatures of Fig. 1B, see supplementary methods. *Pocillopora verrucosa* and *Acropora pulchra* nubbins were collected from a common garden LTER1 site on the back-reef of Paopao Bay, Mo’orea, French Polynesia (17°28'45.0“S 149°50'10.44“W). *Porites rus* nubbins were collected from the fringing reef north of Gump Station research facility (17°29'11.6“S 149°49'31.1“W). Nubbins were collected under permits issued by the French Polynesian Government (Délégation à la Recherche) and the Haut-commissariat de la République en Polynésie Francaise (DTRT) (Protocole d’Accueil 2005-2021). Coral nubbins from the three species were visually inspected at the time of collection and assigned as either “non-bleached” or “bleached” phenotypes. After collection, corals were transported to the Gump Station research facility and acclimated to ambient conditions in a 1300 L flow-through water table for three days. Coral bleaching status was again validated prior to the experiment with a visual inspection and assessment of Symbiodiniaceae cell densities via flow cytometry (Fig. 1C).

#### Pretreatment in flow-through aquaria

At the start of the pre-treatments, coral nubbins were loosely situated in silicon holders and placed into 1.5 L polycarbonate aquaria containing 1.4 L of unfiltered water. To mimic reef-wide bleaching/thermal stress signals, two nubbins from each of the three coral species at a given bleaching phenotype were combined in individual aquaria for a total of six coral fragments in each of the 12 aquaria. Influent water from a flow-through seawater system (sourced from a depth of 6 m directly adjacent to the Gump Station fringing reef) was pumped into the aquaria at a constant rate using a peristaltic pump with platinum cured silicone tubing yielding a final residence time of five hours in the aquaria. Water in each aquaria was recirculated with 4.8 W pumps moving 240 L hr^−1^. Aquaria (*n* = 3 per treatment) were exposed to six days of either ambient (28.6 °C) or elevated water temperatures (32 °C ± 0.2 °C) and ambient light levels (Supplementary Data 2). The water tables holding aquaria were heated using four 300 W and two 800 W Finnex heaters. The combination of bleaching level and temperature yielded four treatments representing a factorial cross of prior bleaching phenotype and temperature: “Control”, “Heated”, “Bleached”, and “Bleached + Heated” (Fig. 1.A.I and 1.A.II). Additionally, two water-only control aquaria, one for each temperature treatment, were included (“Negative Control” and “Negative Control + Heated”).

#### DOM exudation experiment

On the day of the experiment, after seven days of pretreatment, the flow through of unfiltered water and the recirculation of water within the aquaria was stopped. Water was removed from each aquaria until 400 mL remained (roughly 1/3 of the aquaria volume). Subsequently, 800 mL 0.22 µm-filtered offshore water was then added to yield a final volume of 1200 mL. Corals were left in the aquaria to exude DOM for three hours (15:00 h–18:00 h) while heat treatments were maintained (Fig. 1.A.III). After three hours coral community DOM exudates were collected by filtering the 1200 mL of aquaria water through a 0.22 µm PES Sterivex (Millipore) filter into acid-washed 2 L polycarbonate bottles. One of the triplicates of the “Heated” treatment was lost during this step resulting in *n* = 2. To minimize DOM contamination from the filter matrix, all filters were previously flushed with 50–100 mL of 0.22 µm-filtered offshore water. Following exudation corals were removed from the aquaria and airbrushed to collect tissue slurry for downstream Symbiodiniaceae quantification.

#### Dilution cultures

Filtered DOM exudates were used as growth media for dark incubation dilution cultures. Unfiltered back-reef seawater collected from the LTER1 was used as an inoculum. From each replicate aquaria 1200 mL of DOM media was mixed with 400 mL bacterioplankton inoculum (3:1 volumetric ratio) via inversion in acid washed 2 L polycarbonate bottles (Fig. 1.A.IV). Dilution cultures were then split equally into two 1 L acid-washed polycarbonate bottles (800 mL culture per bottle). Half of the bottles were immediately destructively sampled at the beginning of culturing (T0, *n* = 3 per treatment), while the remaining bottles (*n* = 3 per treatment) were incubated in the dark at ambient temperatures for 36 hours.

### Sample collection and processing

#### Symbiodiniaceae quantification

To assess the bleaching status of the corals during collection and at the end of the seven-day incubation and exudation experiment, coral nubbins were flash-frozen and airbrushed using 0.22 µm-filtered seawater. Tissue slurries were analyzed using flow cytometry following the protocol outlined in Fox et al., 2021^85^. For details, please see supplementary methods (Fig. S1).

#### Bacterioplankton abundance

Samples for bacterioplankton abundance measurement via flow cytometry were taken throughout the dilution cultures at 0, 2, 8, 16, 20, 24, 32, and 36 hours. At every time point, 1 mL of each sample was fixed with 16 μL of 32% paraformaldehyde PFA. Samples were run on an Attune Acoustic Focusing Cytometer (Applied Biosystems, Part No. 4445280ASR) at the University of Hawaiʻi at Mānoa to enumerate bacterial cell counts^86^. For sample collection details and flow cytometer settings, see the supplementary methods (Fig. S1).

#### Water collection for bacterial community composition, dissolved organic carbon, and metabolite solid phase extraction

At 0 and 36 h timepoints water (800 mL) was sampled for microbial communities, DOC, and solid phase extraction of DOM using a peristaltic pump connected to acid-washed and seawater-leached silicon tubing. Sample water (800 mL) was passed through a 0.22 µm Sterivex to collect bacterioplankton for downstream DNA analysis.

DOC samples were taken by collecting 35 mL of 0.22 µm sterivex filtrate in acid-washed, combusted, triple sample-rinsed clear glass vials. Care was made to flush each Sterivex with ~50 mL of sample water prior to collecting DOC to avoid contamination from the filter. DOC samples were then acidified with 50 µL of 4 N hydrochloric acid to yield a pH of less than 3. The DOC samples were processed and analyzed via high-temperature combustion on slightly modified Shimadzu TOC-V analyzers at UCSB according to the protocol outlined in Carlson et al. 2010^87^.

For analysis of metabolites, exactly 700 mL of the remaining 0.22 µm Sterivex filtrate was collected in acid-washed 1 L polycarbonate bottles and acidified with HCl to pH < 2. A small volume (50 mL) of the acidified sample water was used to flush the lines prior to the solid phase extraction, resulting in 650 mL of sample for solid phase extraction. Two bottles had less than 650 mL acidified sample water and were equalized to 500 mL solid phase extractions. The difference in volume was later corrected by the resuspension step prior to LC-MS/MS analysis. Metabolites were extracted using a 200 mg mass Bond Elut-PPL (Agilent) cartridges following Dittmar et al. 2008^88^ and Petras et al. 2017^89^. Detailed metabolite extraction methods and all sample handling and storage can be found in the supplementary methods.

#### Microbial community DNA extraction, library prep, and sequencing

Sample DNA extraction protocols followed those outlined in Bullington et al. 2022^90^. For details, please see supplementary methods. Library preparation of the V4 16 S rRNA gene region for amplicon sequencing was conducted at the University of Hawaiʻi at Mānoa Microbial Genomics and Analytical Laboratory using a single barcode library preparation approach with Golay barcoded forward primers and non-barcoded reverse primers. For an overview of primers and settings used, see the supplementary methods. Amplicons were pooled and sequenced using an Illumina MiSeq V3 600 paired-end cycle run at the University of Hawaiʻi at Mānoa Advanced Studies in Genomics, Proteomics, and Bioinformatics facility. A total of 243 samples from this experiment as well as other experiments that occurred at the same field site and time, were included in this sequencing library. All samples were amplified and sequenced in duplicate technical replicates. Method blanks had substantially lower sequence read depth (mean = 1590 reads/sample) than samples (mean = 88,681), with samples ranging from 12,609 reads/sample to 155,685 reads/sample.

#### Dissolved organic matter composition

PPL cartridges were eluted with 2 mL methanol. Extracts were dried down with a vacuum centrifuge and redissolved with 70 µL 80% methanol:water with 1% formic acid. The two samples that had less volume were redissolved to 50 µL so that all concentrations were normalized to filtrate volume. Samples were transferred into a combusted glass insert. A 10 µL aliquot of each sample was analyzed by injection into a Vanquish ultra-high performance liquid chromatography system (UHPLC) coupled to a Q-Exactive Orbitrap Mass Spectrometer (Thermo Fisher Scientific, Bremen, Germany). Chromatographic separation was performed with a C18 core-shell column (Kinetex, 150 × 2 mm, 1.8 µm particle size, 100 Å pore size, Phenomenex, Torrance, USA) all using the settings and protocol described in Petras et al.^89^ and Wegley Kelly et al.^14^.

### Data processing and analysis

#### 16 S amplicon bioinformatics

16 S rRNA gene amplicon sequences were processed using the nextflow bioinformatic pipeline (version 19.10.0) outlined in Arisdakessian et al.^91^ and Jani et al.^92^. Detailed bioinformatic methods can be found in the supplementary methods. In brief, raw paired fastq reads were preprocessed using the DADA2 R package^93^. We used mothur^94^ with the Silva (release 132) database^95^ to align and annotate the sequences, respectively. Per-sample read depth was normalized to 12,000 sequences per sample. OTUs were defined as unique “amplicon sequence variants” (100% clustering OTUs) by DADA2^93^. Lastly, we used the lulu R package to remove artefactual OTUs^96^ and discarded OTUs represented by two or less reads across the 243 samples included in this library. UniFrac distance matrices were constructed from the OTU data and used to assess multivariate differences between microbial communities^97^. At the final time point, two outlier samples were identified and removed from downstream 16 S analysis (outliers were defined as samples whose log10 distance from the centroid of a treatment ≥1.5 SD above the mean log10 distance from the centroid for a given treatment).

#### Metabolomics chemoinformatics

RAW files were converted to .mzML files using MSConvert^98^. MZmine3 (version 3.2.8)^99^ was used for alignment between samples and feature extraction. In order to yield higher consensus alignment quality of MS2 spectra to improve database matching and molecular networking, the 35 samples from this experiment were combined in MzMine with 756 coral reef environmental and experimental DOM samples belonging to tandem studies conducted during the same fieldwork period at Gump Station. Detailed chemoinformatic parameters can be found in the supplemental methods. Metabolite cheminformatics generated 54,040 total molecular ion features (hereafter referred to as features). Ten procedural blanks (LC/MS grade water run in parallel with samples) were included in the run. These procedural blanks were used to identify background features and transient features in the 35 samples from this experiment. Background features were defined as features with an average intensity across all samples which is less than double the maximum intensity of that feature in the procedural blanks. Transient features are defined as features that do not exceed 5 × 10^4^ extracted-ion chromatogram values (XIC) in more than 2 samples. Blank correction and transient feature removal removed 29,286 and 6483 features, respectively. This resulted in 18,271 features with XIC values (Extracted-ion chromatogram values or peak areas), which composes what we consider the exo-metabolome (mixture of ambient and exudate features), which was used in downstream analysis.

#### Statistics

Data analysis and statistics were done using R (version 4.2.1). The main packages used are the core packages within tidyverse^100^, vegan^101^, BiodiversityR^102^, pairwiseAdonis^103^, and stats^104^. The OTU co-occurrence network was generated using SPIEC-EASI^105^ and visualized using Cytoscape (version 3.9.1)^106^. R scripts and additional packages are available through https://github.com/NIOZ-DOM-Analysis/ABCDom.

Symbiodiniaceae cell densities and bacterioplankton cell concentrations had Gaussian distributions after log10 transformation and square root transformation, respectively, and treatment effects were tested using ANOVAs. Surface area normalized DOC flux for the coral treatments was determined by calculating the difference in DOC concentration between each treatment and their respective negative controls, normalizing this value to coral surface area and dividing it by the duration of the DOC exudation period (three hours). DOC areal flux data had a non-Gaussian distribution, and thus, treatment was tested using non-parametric Kruskal-Wallis tests. PERMANOVA tests were run on weighted Unifrac dissimilarity matrices derived from 16 S amplicon sequencing data to test the effect of treatment on microbial community structure. To assess multivariate shifts in the exo-metabolomes, the metabolite feature table containing XIC data was converted to relative abundance data and used to generate a Bray-Curtis dissimilarity matrix, which was then used in downstream PERMANOVA testing. The correlation between metabolomic and microbiota data from this experiment was statistically tested using both Mantel Tests and Procrustes Tests in R (version 4.2.1) and visualized with a Procrustes plot.

### Reporting summary

Further information on research design is available in the Nature Portfolio Reporting Summary linked to this article.

### Supplementary information

Supplementary Information

Description of Additional Supplementary Files

Supplementary Data 1

Supplementary Data 2

Reporting Summary

## Data Availability

Sequencing reads from the demultiplexed samples analyzed in this study have been deposited in the NCBI Sequence Read Archive (SRA) under the BioProject ID: PRJNA1031669. All LC-MS/MS data are publicly available and deposited in the MassIVE data repository (http://massive.ucsd.edu) under the accession number MSV000088021. All data used to generate figures has been deposited in GitHub at https://github.com/NIOZ-DOM-Analysis/ABCDom and are publicly accessioned via Zenodo (10.5281/zenodo.10214648).

## Code availability

Scripts used to analyze the data and generate figures have been deposited in GitHub at https://github.com/NIOZ-DOM-Analysis/ABCDom and are publicly accessioned via Zenodo (10.5281/zenodo.10214648).
