# Supplementary material for: Coral thermal stress and bleaching enrich and restructure reef microbial communities via altered organic matter exudation
Source: Commun Biol. 2024 Feb 13;7:160. doi: 10.1038/s42003-023-05730-0 (PMC10864316; doi:10.1038/s42003-023-05730-0)
Supplement: Supplementary file 1 — Supplementary Information [file 42003_2023_5730_MOESM1_ESM.pdf]

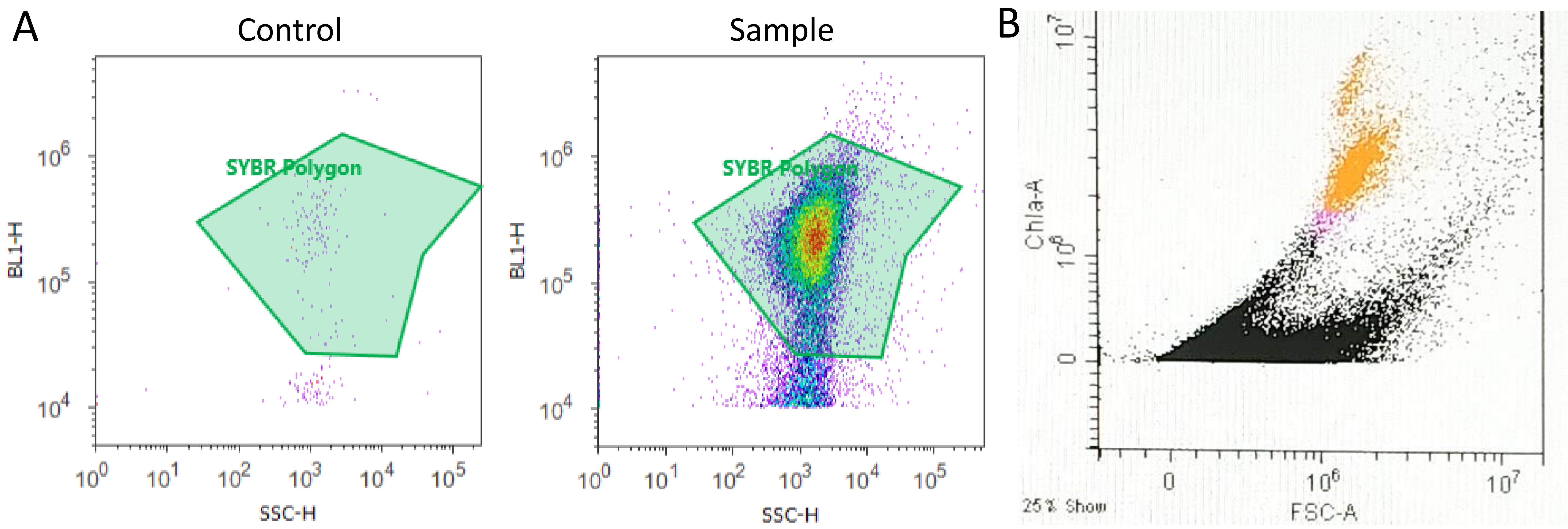

<u>Figure S1</u>: **A)** Representative density plots of gated SYBR polygon derived bacterial counts for a SYBR stained .2µm filtered milliq control and a SYBR stained sample and **B)** a representative dotplot of a gated Symbiodiniaceae population in orange.

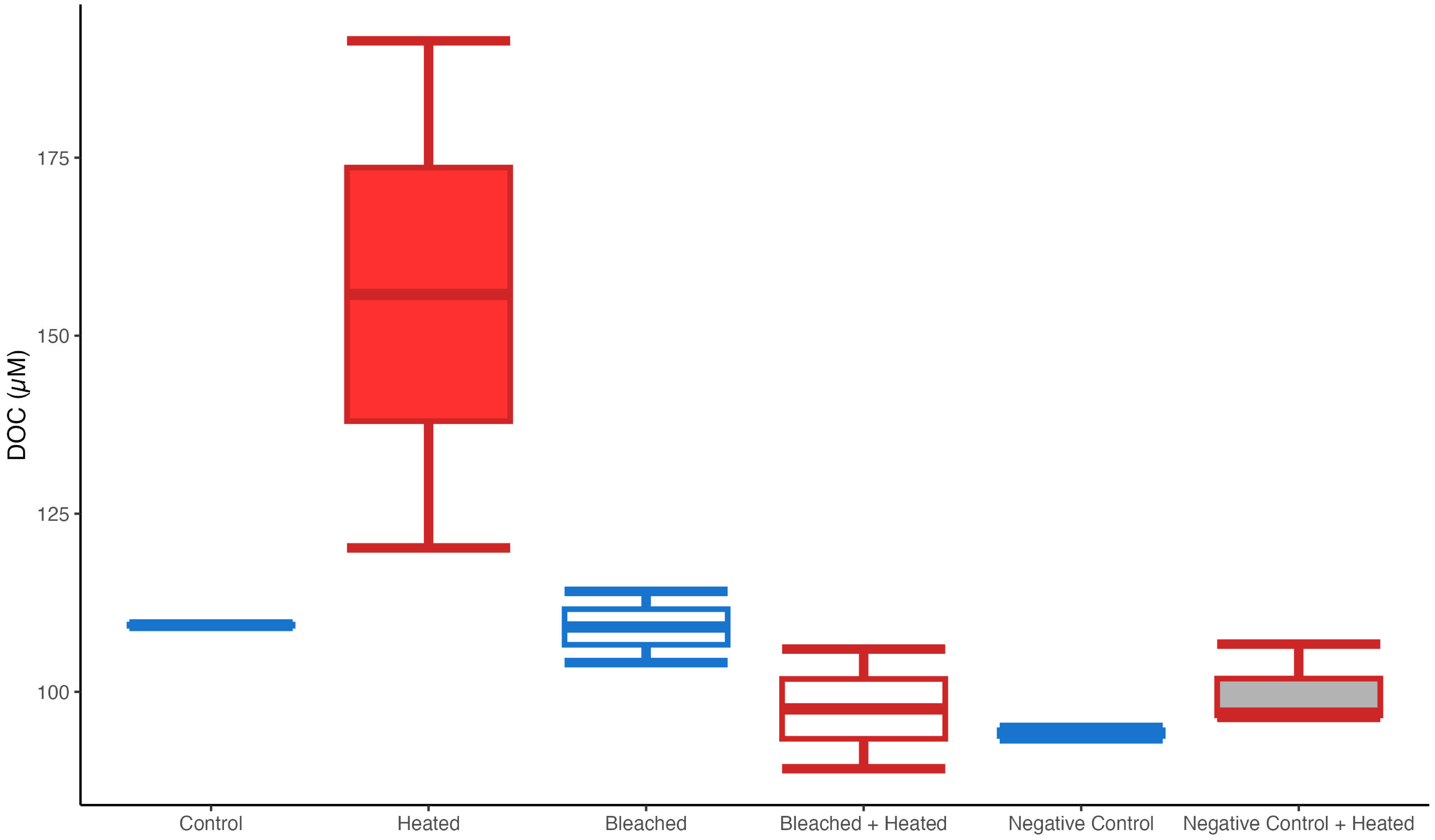

Figure S2: Box and whisker plots of raw DOC exudate concentrations (μM) for the 6 treatments.

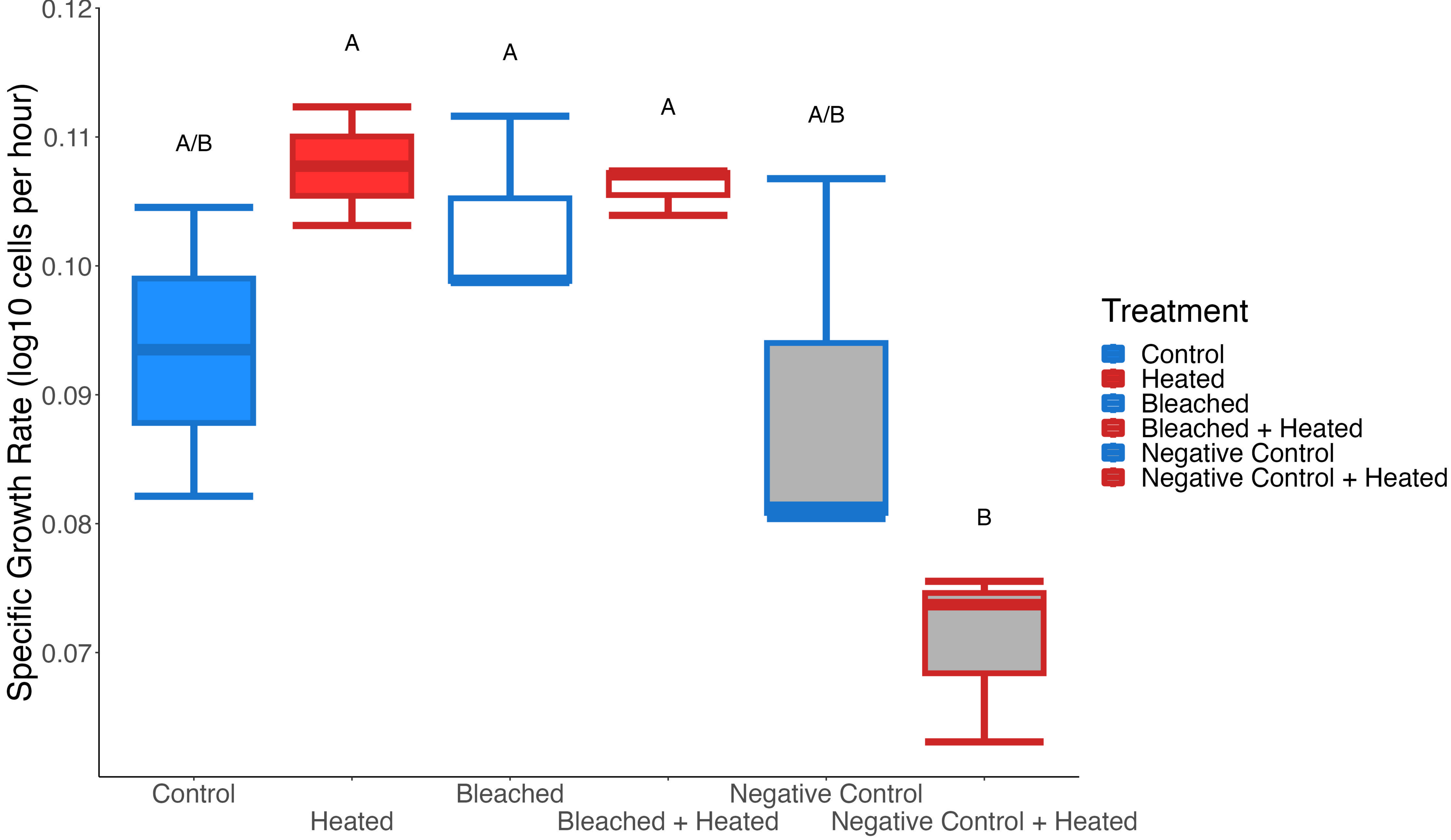

Figure S3: Box and whisker plots of bacterial specific growth rate, in log10 cells per hour, for the 6 treatments. Significant differences between treatments (Tukey post-hoc test, $p<0.05$) are denoted by letters above each boxplot.

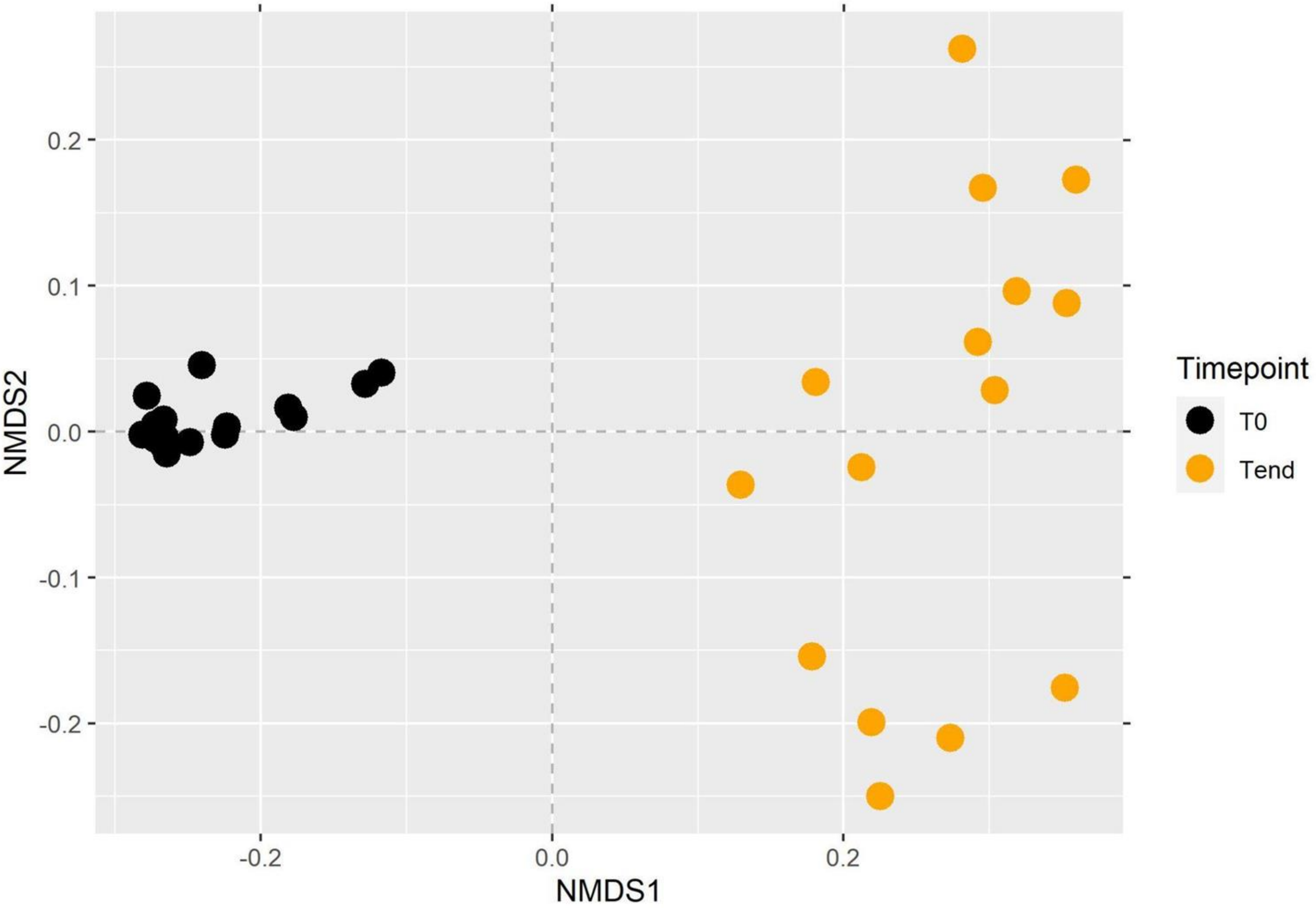

<u>Figure S4</u>: Non-metric multidimensional scaling plot of bacterial communities from start and end of bottle incubation using unifrac dissimilarity.

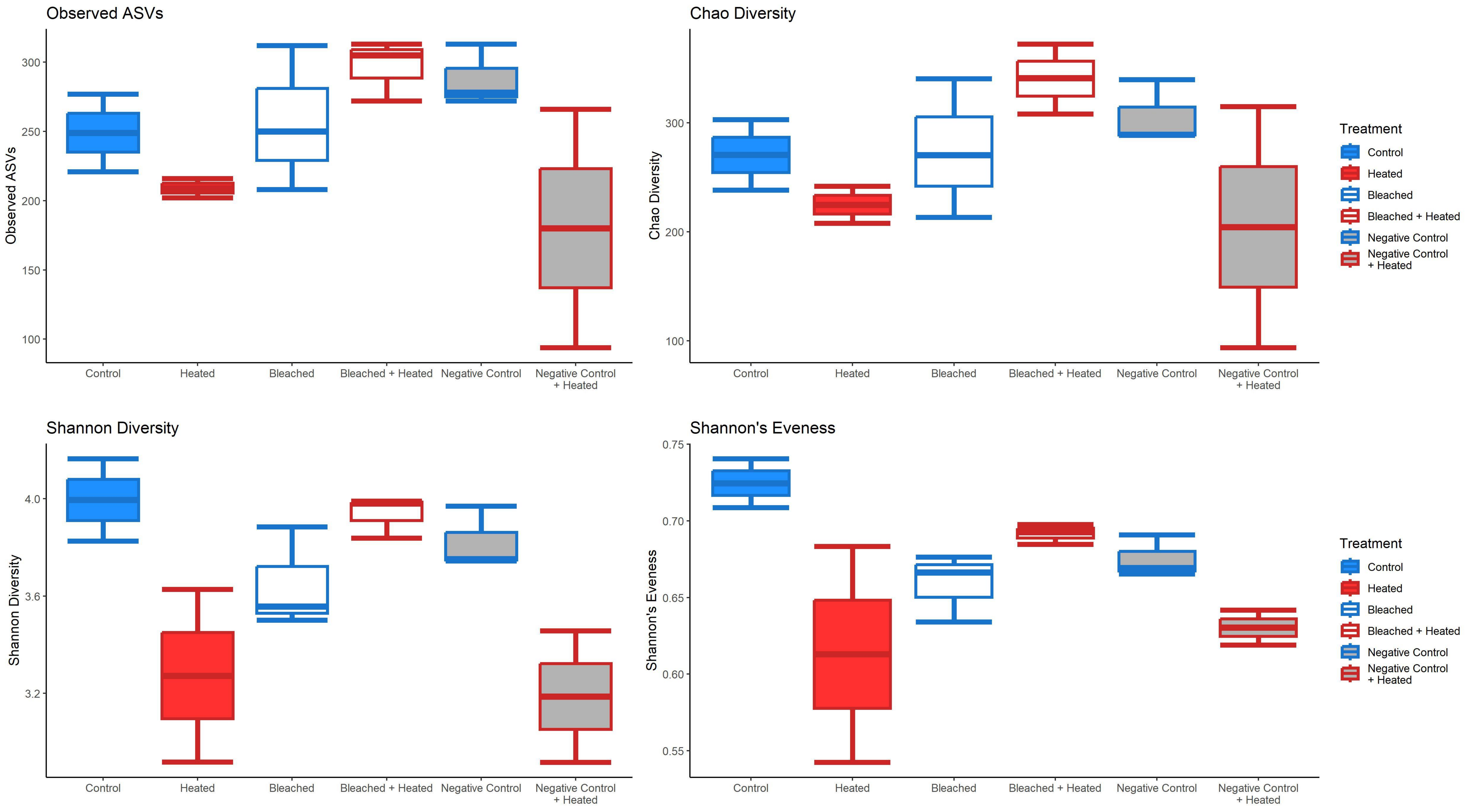

Figure S5: Box and whisker plots of the alpha diversity of the bacterial communities at the end of the incubation

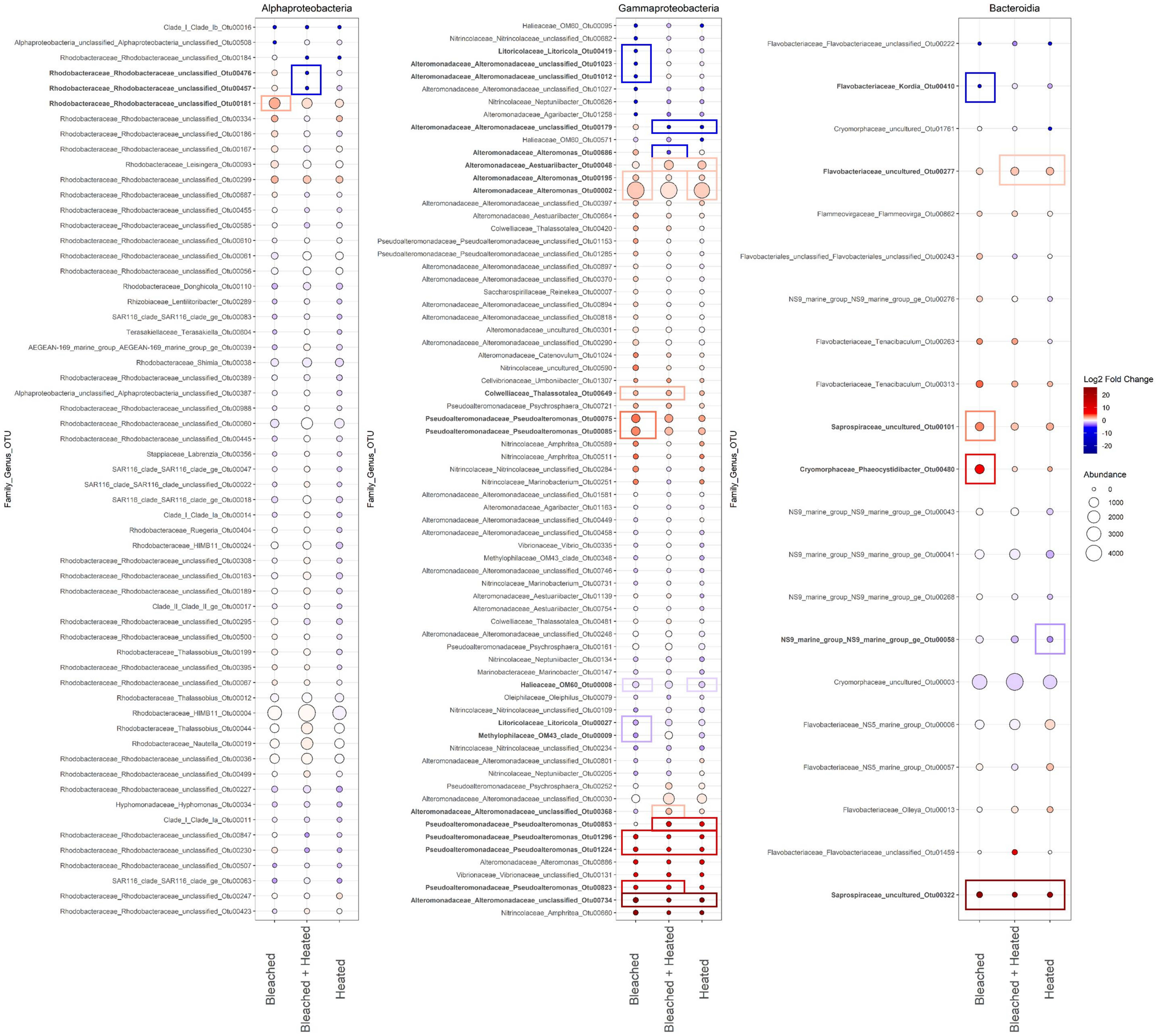

Figure S6: Direct comparison of bacterial OTUs enriched and/or depleted in the three stressed coral treatments relative to the Control corals. The log2 fold change of the 159 most abundant/prevalent OTUs in the three coral stress treatments compared to the Control treatment. Points are colored by log2 fold change, with warmer colors indicating more enrichment and cooler colors indicating more depletion relative to the Controls. Point size indicates the mean abundance of a given OTU in a given treatment. OTUs are labeled according to their family, genus, and OTU Number on the y axis. OTUs labeled in bold were determined by DESEq2 to be significantly differentially abundant in at least one of the three treatments compared to Controls ($p \leq .05$ after FDR). Boxes denote in which treatment there is a significant change and the color of the box indicates whether this was a significant enrichment (red) or depletion (blue).

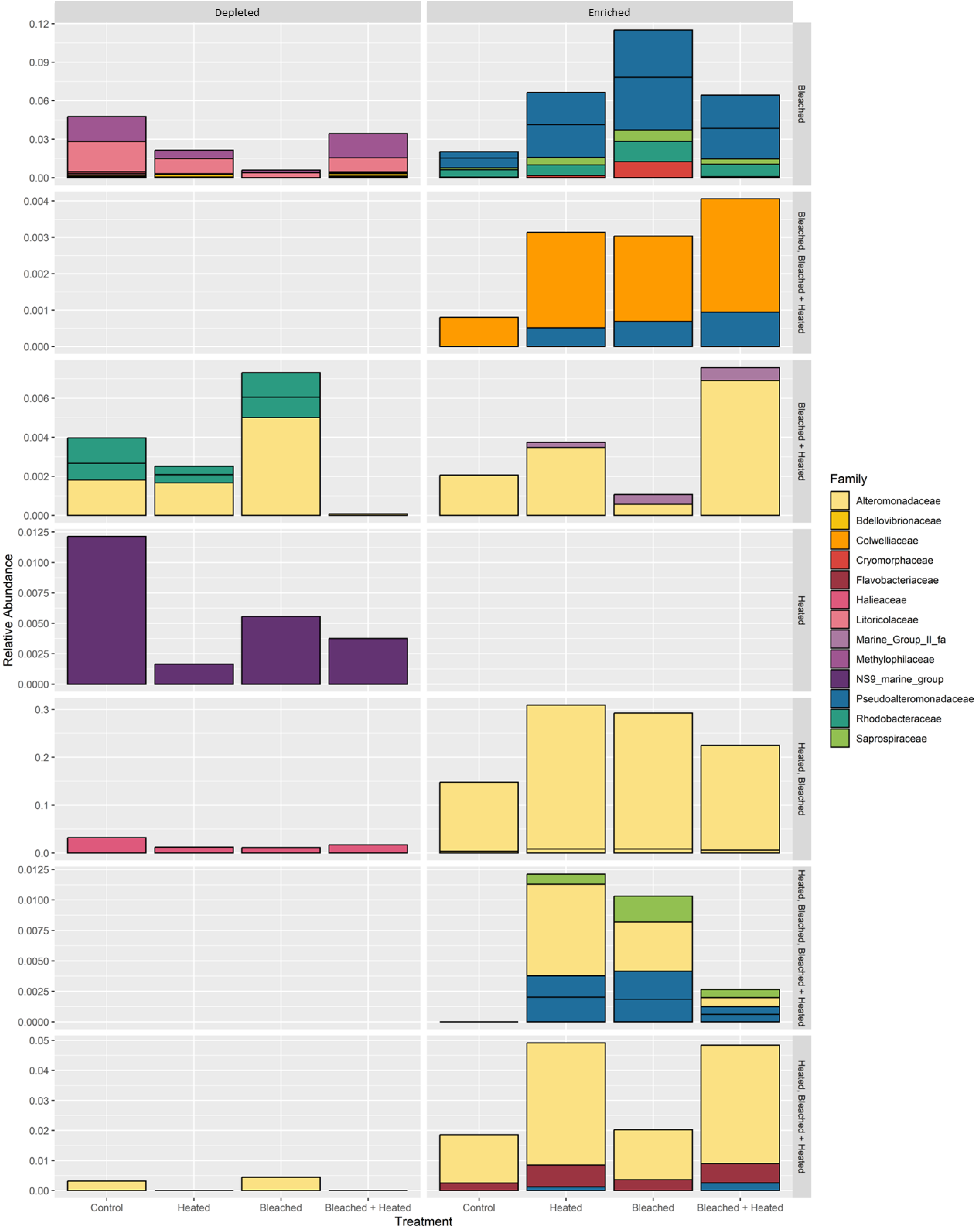

Figure S7: Stacked barplots of the relative abundance of significant OTUs (p≤.05 after FDR) enriched or depleted in any of the 3 coral stress treatments relative to the Control treatment according to DESEq2. Column facets denote if a given OTU is enriched or depleted relative to the Control. Row facets denote which treatments a group of OTUs is either significantly enriched or depleted in. Relative abundance was derived from the non-subsampled, raw abundance data used in DESEq2. Bars are colored according to bacterial family.

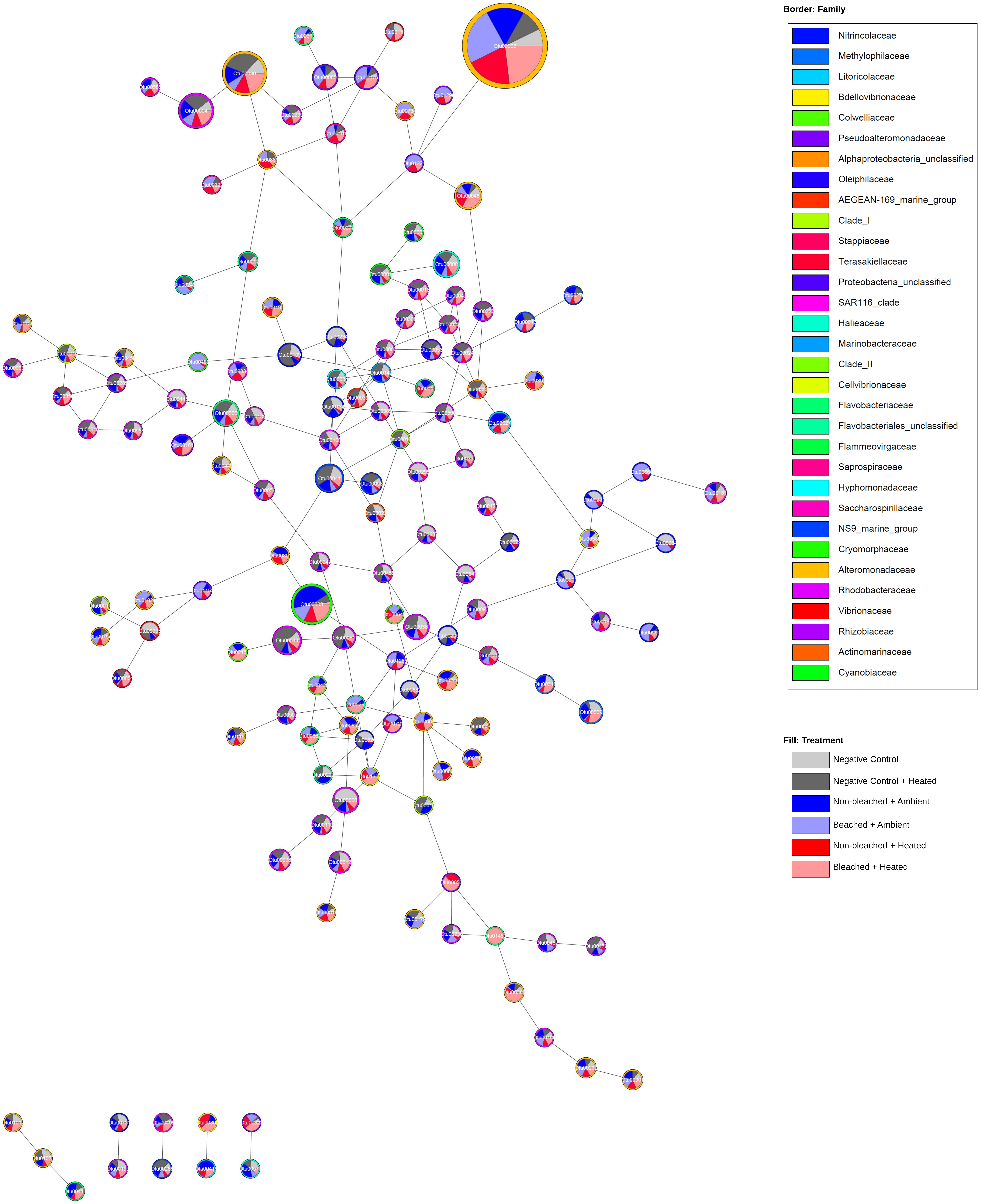

<u>Figure S8:</u> Cooccurence network of microbial OTUs generated from SpiecEasi and visualized using Cytoscape. Vertices designated significant positive pairwise correlations as defined by SpiecEasi. Nodes represent individual OTUs. Node size indicates mean abundance of a given OTU, node outline is colored according to OTU family, and node fill corresponds to the relative proportion of a given OTU in each of the 6 treatments.

SUPPLEMENTARY METHODS

Temperature Trends

Prior to sampling there were multiple thermal anomalies starting in December 2018 in which temperatures exceeded the thermal stress accumulation threshold level of 29 °C (Leinbach et al., 2021; Pratchett et al., 2013; Speare et al., 2021) by >1°C. Temperature trends were analyzed using the Mo'orea Coral Reef Long Term Ecological Research (MCR LTER) daily average water temperature data. Time-series data were collected from 3 sites on the MCR LTER fore-reef: FOR1, FOR4 and FOR5 (GPS location: 17°28'30.0"S 149°50'13.2"W; 17°32'49.2"S 149°46'08.4"W; 17°34'55.2"S 149°52'30.0"W; respectively). From each location, measurements from five sensors ("upper water column", "middle water column", "bottom water column", "temperature shallow", and "temperature deeper") was used to calculate the average temperature +/- one standard deviation. Bleaching was first observed in the corals adjacent to Gump Station, Mo'orea, in April 2019 (Leinbach et al., 2021) (Figure 1B). Accumulated degree heating days reached a maximum of 17°C-Days in mid-April before rapidly decreasing (Burgess et al., 2021). By the start of field collection on May 8th, 2019, the temperatures dropped below the 29 °C threshold. Corals had experienced a total of 110 days of temperatures exceeding the threshold in a period of five months (151 days).

Bacterioplankton Abundance and Flowcytometry Settings

Samples for bacterioplankton abundance were flash-frozen at -40 °C for 14 days prior to transportation to University of Hawaiʻi at Mānoa campus where they were frozen at -80 °C for six months prior to flow cytometry processing. Fixed microbial abundance samples were thawed, and 200 µL of each sample aliquoted and stained with 2 µL 100X SYBR Green to be run on an Attune Acoustic Focusing Cytometer (Applied Biosystems, Part No. 4445280ASR) at University of Hawaiʻi at Mānoa to enumerate bacterial cell counts.

The BL1 detector for the blue laser (488 nm) and the SSC detector for the violet laser (405 nm) were used in conjunction to elucidate bacterial abundances. Voltages and gating were manually determined to enable easy identification of SYBR green stained bacterioplankton populations using a BL1 voltage of 2,625 mV and a SSC voltage of 2,500 mV. Density plots of BL1 vs. SSC were gated on the easily distinguishable population of SYBR green stained bacteria (Figure S1a).

Symbiodiniaceae Quantification

Samples were frozen at -40 °C for 14 days prior to transportation to University of Hawaiʻi at Mānoa campus where they were frozen at -80 °C for 23 months prior to flow cytometry processing. In brief, samples were thawed, briefly homogenized using a vortex machine, and 200 µL of each sample being aliquoted into 96-well round-bottom flow cytometry plates. Symbiodiniaceae slurries were run on a Beckman Coulter CytoFLEX S Flow Cytometer (Beckman Coulter, Product No: B78560).

The Chlorophyll-a emissions PMT and the two scatter detectors for the violet laser were used in conjunction to count Symbiodiniaceae cells, delineated as distinct populations of large cells with high specific *chlorophyll a* content. Voltages and gating were manually determined to enable easy identification of this population using a FSC gain of 100, a SSC gain of 100, and a Chla gain of 50. Chla values had a lower threshold of 20,000. Density plots of Chla vs. FSC were gated on the easily distinguishable population of SYBR green stained bacteria (Figure S1b).

Metabolomics Sample Collection

Prior to sampling, 200 mg mass Bond Elut-PPL (Agilent) cartridges were soaked overnight in LC-MS grade methanol followed by a series of washes (2x washes of methanol, 2x washes of LC-MS grade water, 1x wash of methanol) before air-drying the cartridges. In the

field, PPL cartridges were activated with 1x methanol wash followed by 2x pH 2 LC-MS grade water washes. Samples were loaded on the cartridges by pumping the acidified filtrate over the PPL cartridges at 8mL/min. Cartridges were desalinated using a 3x pH2 LC-MS grade water wash and dried using N_2 gas.

Sample Storage for Bacterial Community Composition, Dissolved Organic Carbon and Metabolite Solid Phase Extraction

Sterivex filters were frozen at -40 °C for 14 days prior to transportation to University of Hawaiʻi at Mānoa campus where they were transferred to -80 °C for six months prior to DNA extraction. DOC samples were kept at room temperature for 14 days prior to transportation to the University of California, Santa Barbara (UCSB). At UCSB the DOC samples were stored at 15 °C in the dark for six months prior to sample processing. PPL Cartridges were stored at -80 °C until samples were processed at UCSD in spring 2021.

Microbial Community DNA Extraction, Library Prep, and Sequencing

To extract bacterial DNA from the Sterivex filters, the filter was removed from the plastic casing using sterile pliers, scalpels, and tweezers, and added to MP Biomedicals Lysing Matrix A (No. 116910100) tubes with 0.5 mL MC 1 lysis buffer and homogenized using a MP Biomedicals FastPrep-96 bead beater. A portion of the homogenate (0.4 mL) was recovered and DNA extractions were completed using the Macherey-Nagel NucleoMag Plant Extraction Kit (No. 744400.4) with KingFisher Accessory Kit (No. 744951). DNA samples were eluted to a final volume of 110 µL.

515F (Parada et al., 2016) and 806R (Apprill et al., 2015) Earth Microbiome Project primers were used according to Walters et al., 2016 with barcodes on the 515F primer. Amplicons were generated from a single round of PCR using primers that include Illumina spacers, Illumina adapters, index sequences (on the forward primers), and 16S rRNA gene

template region. PCR reagents included 7.2 µl DNase free water, 10 µl PLAT II MM (2X) (Invitrogen, Platinum II Hot-Start PCR Master Mix, Catalog No: 14000012), 0.4 µl forward primer (10 µM), 0.4 µl reverse primer (10 µM), and 2 µl DNA template. PCR was performed on an Applied Biosystems SimpliAmp (Catalog No: A24811) system using an initial denature of 94 °C for 2:00 min followed by 35 cycles of 94 °C for 15 s, 54 °C for 15 s, 68 °C for 7 s, followed by a final extension at 68 °C for 3 min. DNA extraction blanks and no-template control blanks were included as negative controls.Mock communities (ZymoBIOMICS Microbial Community DNA Standard, Cat No: D6305) were included as positive controls to detect contaminants from kits or library preparation. Total amplicons per sample were normalized to between 12.5 and 15 ng using Charm Biotech Just-a-Plate PCR purification and normalization kit.

16 Amplicon Bioinformatics

Raw paired fastq reads were preprocessed using the DADA2 R package (Callahan et al., 2016a). We truncated forward reads at position 220 and reverse reads at position 190 and discarded them if they contained a number of expected errors above three using the filterAndTrim() function. Denoising was performed with the learnError() and dada() functions with default parameters. Using the mergePairs() function, we merged reads if they overlapped by at least 20 bases, and allowed for 1 mismatch at most. Duplicate technical replicates were then merged bioinformatically. We used mothur (Schloss et al., 2009) along with the Silva (release 132) database (Quast et al., 2013) to align and annotate the sequences, respectively. Sequences with a start or stop position outside the 5th-95th percentile range (over all sequences) were discarded. We removed potential chimeras with chimera.vsearch(). Taxonomies were assigned using classify.seqs() and classify.otus(). We removed all mitochondrial or chloroplast OTUs, as well as sequences with no annotations at the domain level. Using sub.sample(), we normalized the abundance in each sample by subsampling to 12,000 sequences. OTUs were defined as unique “amplicon sequence variants” (100%

clustering OTUs) by DADA2 (Callahan et al., 2016). We used the lulu R package to remove artefactual OTUs (Frøslev et al., 2017): we merged two OTUs if all of the 3 following conditions were satisfied: 1) They co-occur in every sample, 2) One of the two OTUs has a lower abundance than the other in every sample and 3) they share a sequence similarity of at least 97%. Finally, we discarded OTUs represented by two or less reads across the 243 samples included in this library. UniFrac distance matrices were constructed from the OTU data and used to assess multivariate differences between microbial communities (Lozupone & Knight, 2005). At the final time point, two outlier samples were identified and removed from downstream 16S analysis (outliers were defined as samples whose log10 distance from the centroid of a treatment ≥ 1.5 SD above the mean log10 distance from the centroid for a given treatment).

Metabolomics Chemoinformatic Methods

Untargeted LC-MS/MS data pre-processing was performed with MzMine3 v3.2.8 (Pluskal et al., 2010). Mass detection was performed using the “centroid” algorithm. Intensity thresholds of 1E5 and 1E3 were for used for MS1 and MS2, respectively. Chromatograms were built using the ADAP chromatogram builder with a min group size of four, group intensity threshold of 2E5, minimum peak intensity of 1E5, and m/z tolerance of 0.0015 Da or 10 ppm. Extracted Ion Chromatograms (XICs) were deconvoluted using the local minimum search algorithm with a chromatographic threshold of 85% , a search minimum in RT range of 0.08 min, and a median m/z center calculation with m/z range for MS2 pairing of 0.1 and RT range for MS2 scan pairing of 0.15. Isotope peaks were grouped and features from different samples were aligned with 0.001 Da or 5 ppm mass tolerance and 0.1 min retention time tolerance. MS1 peak lists were joined using an m/z tolerance of 0.0015 Da or 10 ppm and retention time tolerance of 0.15 min. Alignment was then performed by placing a weight of one on RT and a mobility weight of one. The feature table of peak areas were exported as a .csv file and the corresponding consensus MS/MS spectra were exported as a .mgf file.

Bacterial Differential Abundance Analysis

In order to directly elucidate which specific bacterial taxa were driving these differences, we performed DESeq2, a method for analysis of differential expression of count data derived from high throughput sequencing, on a subset of the data that only included the four coral DOM treatments (Love et al., 2014). DESeq2 requires raw read inputs prior to reads per sample normalization and was thus run on raw read counts prior to the subsampling and Lulu steps of our bioinformatic pipeline. In order to eliminate low abundance and prevalence of OTUs prior to DESeq2, OTUs were removed so that only those with raw abundance ≥ 50 in three or more samples or a raw abundance ≥ 1000 in one or more samples were included, which comprised a subset of 187 OTUs. Given that OTU abundances in the stressed coral treatments were going to be compared to the coral controls, we further removed 28 highly variable OTUs within the Ccontrol coral treatment. Specifically, all OTUs with a coefficient of variation (CV) greater than 1 standard deviation of the mean CV of all OTUs were culled, yielding a final count of 159 OTUs to be run through DESeq2.

SUPPLEMENTARY REFERENCES

Apprill, A., McNally, S., Parsons, R., & Weber, L. (2015). Minor revision to V4 region SSU rRNA 806R gene primer greatly increases detection of SAR11 bacterioplankton. *Aquatic Microbial Ecology*, *75*(2), 129–137. https://doi.org/10.3354/ame01753

Leinbach, S. E., Speare, K. E., Rossin, A. M., Holstein, D. M., & Strader, M. E. (2021). Energetic and reproductive costs of coral recovery in divergent bleaching responses. *Scientific Reports*, *11*(1), Article 1. https://doi.org/10.1038/s41598-021-02807-w

Love, M. I., Huber, W., & Anders, S. (2014). Moderated estimation of fold change and dispersion for RNA-seq data with DESeq2. *Genome Biology*, *15*(12), 550. https://doi.org/10.1186/s13059-014-0550-8

Parada, A. E., Needham, D. M., & Fuhrman, J. A. (2016). Every base matters: Assessing small subunit rRNA primers for marine microbiomes with mock communities, time series and global field samples. *Environmental Microbiology*, *18*(5), 1403–1414. https://doi.org/10.1111/1462-2920.13023

Pluskal, T., Castillo, S., Villar-Briones, A., & Oresic, M. (2010). MZmine 2: Modular framework for processing, visualizing, and analyzing mass spectrometry-based molecular profile data. *BMC Bioinformatics*, *11*, 395. https://doi.org/10.1186/1471-2105-11-395

Pratchett, M. S., McCowan, D., Maynard, J. A., & Heron, S. F. (2013). Changes in Bleaching Susceptibility among Corals Subject to Ocean Warming and Recurrent Bleaching in Moorea, French Polynesia. *PLOS ONE*, *8*(7), e70443. https://doi.org/10.1371/journal.pone.0070443

Speare, K. E., Adam, T. C., Winslow, E. M., Lenihan, H. S., & Burkepile, D. E. (2021). Size-dependent mortality of corals during marine heatwave erodes recovery capacity of a coral reef. *Global Change Biology*, *28*(4), 1342–1358. https://doi.org/10.1111/gcb.16000

Walters, W., Hyde, E. R., Berg-Lyons, D., Ackermann, G., Humphrey, G., Parada, A., Gilbert, J. A., Jansson, J. K., Caporaso, J. G., Fuhrman, J. A., Apprill, A., & Knight, R. (n.d.). Improved

Bacterial 16S rRNA Gene (V4 and V4-5) and Fungal Internal Transcribed Spacer Marker Gene Primers for Microbial Community Surveys. *MSystems*, *1*(1), e00009-15. https://doi.org/10.1128/mSystems.00009-15

Family	Ambient Water Control	Bleached	Bleached + Heated	Control	Negative Control	Negative Control + Heated
**Alteromonadaceae**	0.226792	0.425707	0.386279	0.283001	0.466167	0.203806
**Bdellovibrionaceae**	0.001584	0.002792	0.051251	0.022333	0.039083	0.001723
**Clade_I**	0.004751	0.001125	0.002584	0.004917	0.001375	0.006055
**Colwelliaceae**	0.001875	0.0175	0.012389	0.011139	0.011042	0.000472
**Cryomorphaceae**	0.007875	0.072083	0.057972	0.129278	0.056333	0.017111
**Cyanobiaceae**	0.010042	0.004958	0.004917	0.01	0.004751	0.017055
**Flavobacteriaceae**	0.039043	0.036251	0.044833	0.04436	0.082708	0.048277
**Flavobacteriales_unclassified**	0.005668	0.007458	0.009555	0.012027	0.004375	0.007223
**Halieaceae**	0.028792	0.013	0.0195	0.038306	0.012625	0.023194
**Litoricolaceae**	0.003833	0.003583	0.011528	0.025444	0.011625	0.002305
**Marine_Group_II_fa**	0.005834	0.002875	0.005083	0.001306	0.001333	0.008278
**Marinobacteraceae**	0.004542	0.000917	0.001917	0.003556	0.00075	0.005694
**Methylophagaceae**	0.006792	0.006125	0.004305	0.005306	0.01475	0.0455
**Methylophilaceae**	0.020417	0.002792	0.020583	0.022	0.006917	0.002861
**Nitrincolaceae**	0.111748	0.043625	0.01425	0.027361	0.037417	0.055972
**NS9_marine_group**	0.04225	0.028833	0.030028	0.047695	0.01125	0.06925
**Oleiphilaceae**	0.002459	0.000292	5.00E-04	0.005528	0.000417	0.005695
**Proteobacteria_unclassified**	0.004042	0.002458	0.002972	0.006833	0.002375	0.0095
**Pseudoalteromonadaceae**	0.035792	0.122251	0.08125	0.033472	0.070583	0.022223
**Rhodobacteraceae**	0.380831	0.166	0.194276	0.212747	0.136707	0.333888
**Saprospiraceae**	0.002334	0.013083	0.005584	0.002055	0.007542	0.003832
**SAR116_clade**	0.008167	0.00425	0.009638	0.013028	0.003292	0.016917
**Vibrionaceae**	0.011084	0.00325	0.002806	0.002445	0.002459	0.004944
**Other**	0.033459	0.018797	0.026	0.035864	0.014128	0.088225

Table S1: Mean relative abundance of dominant Families in the 6 treatments
