# Supplementary material for: Coral thermal stress and bleaching enrich and restructure reef microbial communities via altered organic matter exudation
Source: Commun Biol. 2024 Feb 13;7:160. doi: 10.1038/s42003-023-05730-0 (PMC10864316; doi:10.1038/s42003-023-05730-0)
Supplement: Supplementary file 2 — Description of Additional Supplementary Files [file 42003_2023_5730_MOESM2_ESM.pdf]

Description of Additional Supplementary Files

File name: Supplementary Data 1
Description: DESEq2 results for the 159 OTUs whose differential abundance was tested between the three coral stress treatments and the coral controls.

File name: Supplementary Data 2
Description: High temporal resolution temperature and light intensity data throughout the flow-through aquaria incubation for the ambient temperature water table. Point temperature measurements for the heated water table throughout the incubation. Measurements are labeled according to which portion of the experiment they were taken during (acclimatization, etc.)
