# Supplementary material for: Coral thermal stress and bleaching enrich and restructure reef microbial communities via altered organic matter exudation
Source: Commun Biol. 2024 Feb 13;7:160. doi: 10.1038/s42003-023-05730-0 (PMC10864316; doi:10.1038/s42003-023-05730-0)
Supplement: Supplementary file 5 — Reporting Summary [file 42003_2023_5730_MOESM5_ESM.pdf]

nature portfolio

Corresponding author(s): Wesley Sparagon
Last updated by author(s): 2023 August 30

Reporting Summary

Nature Portfolio wishes to improve the reproducibility of the work that we publish. This form provides structure for consistency and transparency in reporting. For further information on Nature Portfolio policies, see our Editorial Policies and the Editorial Policy Checklist.

Statistics

For all statistical analyses, confirm that the following items are present in the figure legend, table legend, main text, or Methods section.

n/a	Confirmed	
☐	☒	The exact sample size (*n*) for each experimental group/condition, given as a discrete number and unit of measurement
☐	☒	A statement on whether measurements were taken from distinct samples or whether the same sample was measured repeatedly
☐	☒	The statistical test(s) used AND whether they are one- or two-sided<br>*Only common tests should be described solely by name; describe more complex techniques in the Methods section.*
☐	☒	A description of all covariates tested
☐	☒	A description of any assumptions or corrections, such as tests of normality and adjustment for multiple comparisons
☐	☒	A full description of the statistical parameters including central tendency (e.g. means) or other basic estimates (e.g. regression coefficient) AND variation (e.g. standard deviation) or associated estimates of uncertainty (e.g. confidence intervals)
☐	☒	For null hypothesis testing, the test statistic (e.g. *F*, *t*, *r*) with confidence intervals, effect sizes, degrees of freedom and *P* value noted<br>*Give P values as exact values whenever suitable.*
☒	☐	For Bayesian analysis, information on the choice of priors and Markov chain Monte Carlo settings
☒	☐	For hierarchical and complex designs, identification of the appropriate level for tests and full reporting of outcomes
☒	☐	Estimates of effect sizes (e.g. Cohen's *d*, Pearson's *r*), indicating how they were calculated

Our web collection on statistics for biologists contains articles on many of the points above.

Software and code

Policy information about availability of computer code

Data collection	No software was used for data collection in this study.
Data analysis	16S amplicon bioinformatics were performed using the nextflow bioinformatic pipeline (version 19.10.0) outlined in Arisdakessian et al., 2020 and Jani et al., 2021. This included the use of the DADA2 R package (Callahan et al., 2016), mothur (Schloss et al., 2009) with the Silva (release 132) database (Quast et al., 2013), and the lulu R package (Frøslev et al., 2017). Chemoinformatics used MSConvert (Chambers et al., 2012) and MZmine3 (version 3.2.8) (Pluskal et al., 2010) to process metabolomic data. Statistics and visualizations were done using R (version 4.2.1). Main packages used are the core packages within tidyverse (Wickham et al., 2019), vegan (Oksanen, 2013), BiodiversityR (Kindt & Coe, 2005), pairwiseAdonis (Martinez P., 2020), and stats (R Core Team, 2013). The OTU co-occurrence network was generated using SPIEC-EASI (Kurtz et al., 2015) and visualized using Cytoscape (version 3.9.1) (Shannon et al., 2003). Scripts used to analyze the data in R have been deposited in GitHub at https://github.com/NIOZ-DOM-Analysis/ABCDom (repository made public with DOI via Zenodo upon acceptance).

For manuscripts utilizing custom algorithms or software that are central to the research but not yet described in published literature, software must be made available to editors and reviewers. We strongly encourage code deposition in a community repository (e.g. GitHub). See the Nature Portfolio guidelines for submitting code & software for further information.

Data

Policy information about availability of data

All manuscripts must include a data availability statement. This statement should provide the following information, where applicable:

- Accession codes, unique identifiers, or web links for publicly available datasets
- A description of any restrictions on data availability
- For clinical datasets or third party data, please ensure that the statement adheres to our policy

Sequencing reads from the demultiplexed samples analyzed in this study have been deposited in the NCBI Sequence Read Archive (SRA) under the BioProject accession xxxxxx (submission to SRA to be finalized and made public upon acceptance). All LC-MS/MS data are publicly available and deposited in the MassIVE data repository (http://massive.ucsd.edu) under the accession number MSV000088021 (MassIVE repository made public upon acceptance).

Human research participants

Policy information about studies involving human research participants and Sex and Gender in Research.

Reporting on sex and gender	*Use the terms sex (biological attribute) and gender (shaped by social and cultural circumstances) carefully in order to avoid confusing both terms. Indicate if findings apply to only one sex or gender; describe whether sex and gender were considered in study design whether sex and/or gender was determined based on self-reporting or assigned and methods used. Provide in the source data disaggregated sex and gender data where this information has been collected, and consent has been obtained for sharing of individual-level data; provide overall numbers in this Reporting Summary. Please state if this information has not been collected. Report sex- and gender-based analyses where performed, justify reasons for lack of sex- and gender-based analysis.*
Population characteristics	*Describe the covariate-relevant population characteristics of the human research participants (e.g. age, genotypic information, past and current diagnosis and treatment categories). If you filled out the behavioural & social sciences study design questions and have nothing to add here, write "See above."*
Recruitment	*Describe how participants were recruited. Outline any potential self-selection bias or other biases that may be present and how these are likely to impact results.*
Ethics oversight	*Identify the organization(s) that approved the study protocol.*

Note that full information on the approval of the study protocol must also be provided in the manuscript.

Field-specific reporting

Please select the one below that is the best fit for your research. If you are not sure, read the appropriate sections before making your selection.

☐ Life sciences ☐ Behavioural & social sciences ☒ Ecological, evolutionary & environmental sciences

For a reference copy of the document with all sections, see nature.com/documents/nr-reporting-summary-flat.pdf

Ecological, evolutionary & environmental sciences study design

All studies must disclose on these points even when the disclosure is negative.

Study description	A mesocosm heating experiment and bottle incubation compared how unbleached and bleached corals alter dissolved organic matter (DOM) exudation in response to thermal stress and subsequent effects on microbial growth and community structure in the water column. In brief, coral nubbins from three different species (Pocillopora verrucosa, Acropora pulchra, and Porites rus) assigned to both bleached and unbleached phenotypes were collected and exposed to six days of either ambient (28.6 °C) or elevated water temperatures (32 ℃ +/- 0.2 ℃) and ambient light levels in flow through aquaria (n=3 per treatment). The combination of bleaching level and temperature yielded four treatments representing a factorial cross of prior bleaching phenotype and temperature: “Control”, “Heated”, “Bleached”, and “Bleached + Heated” (Fig 1 A.II). Additionally, two water-only control aquaria, one for each temperature treatment, were included (“Negative Control” and “Negative Control + Heated”). DOM was collected from these 6 treatments, analyzed, and fed to ambient bacterioplankton communities in dark bottle incubations for 36 hours. These 6 treatments were considered as 6 different levels of a single factor for downstream statistical analysis.
Research sample	To mimic reef-wide bleaching/thermal stress signals, two nubbins from each of three coral species common in Mo'orea (Pocillopora verrucosa, Acropora pulchra, and Porites rus) at a given bleaching phenotype were combined with unfiltered water in individual aquaria for a total of six coral fragments in each of the 12 aquaria. Aquaria (n=3 per treatment) were exposed to six days of either ambient (28.6 °C) or elevated water temperatures (32 ℃ +/- 0.2 ℃) and ambient light levels. Rather than test for species-specific differences in DOM release and bacterioplankton response, we opted to combine the 3 coral species in individual aquaria to mimic the natural composition of coral communities on Mo'orea (Edmunds, 2022) and assess the general coral community response to thermal stress and bleaching.

Sampling strategy

Sample sizes were chosen based on physical constraints of the water tables and aquaria. These samples sizes were deemed to be sufficient because studies with very similar experiment designs, procedures, and datasets (Wegley Kelly et al., 2022; Nelson et al., 2013) used comparable sample sizes. On the day of the experiment, after seven days of pretreatment, the flow through of unfiltered water and the recirculation of water within the aquaria was stopped. Water was removed from each aquaria until 400 mL remained (roughly ⅓ of the aquaria volume). Subsequently, 800 mL 0.22 µm-filtered offshore water was then added to yield a final volume of 1200 mL. Corals were left in the aquaria to exude DOM for three hours (15:00 h - 18:00 h) while heat treatments were maintained (Fig 1.A.III). After three hours coral community DOM exudates were collected by filtering the 1200 mL of aquaria water through a 0.22 µm PES Sterivex (Millipore) filter into acid-washed 2 L polycarbonate bottles. One of the triplicates of the "Heated" treatment was lost during this step resulting in n=2. To minimize DOM contamination from the filter matrix, all filters were previously flushed with 50-100 mL of 0.22 µm filtered offshore water. Following exudation corals were removed from the aquaria and airbrushed to collect tissue slurry for downstream Symbiodiniaceae quantification.

Dilution Cultures
Filtered DOM exudates were used as growth media for dark incubation dilution cultures. Unfiltered back-reef seawater collected from the LTER 1 was used as an inoculum. From each replicate aquaria 1200 mL of DOM media was mixed with 400 mL bacterioplankton inoculum (3:1 volumetric ratio) via inversion in acid washed 2 L polycarbonate bottles (Figure 1.A.IV). Dilution cultures were then split equally into two 1 L acid washed polycarbonate bottles (800 mL culture per bottle). Half of the bottles were immediately destructively sampled at the beginning of culturing (T0, n=3 per treatment), while the remaining bottles (n=3 per treatment) were incubated in the dark at ambient temperatures for 36 hours.

Sample Collection and Processing
Symbiodiniaceae Quantification
To assess bleaching status of the corals during collection and at the end of the seven day incubation and exudation experiment, coral nubbins were flash-frozen and airbrushed using 0.22 µm filtered seawater. Tissue slurries were analyzed using flow cytometry following the protocol outlined in Fox et al., 2021.

Bacterioplankton Abundance
Samples for bacterioplankton abundance measurement via flow cytometry were taken throughout the dilution cultures at 0, 2, 8, 16, 20, 24, 32, and 36 hours. At every time point, 1 mL of each sample was fixed with 16 µL of 32% paraformaldehyde PFA. Samples were run on an Attune Acoustic Focusing Cytometer (Applied Biosystems, Part No. 4445280ASR) at University of Hawaiʻi at Mānoa to enumerate bacterial cell counts (Nelson et al., 2015).

Water Collection for Bacterial Community Composition, Dissolved Organic Carbon and Metabolite Solid Phase Extraction
At 0 and 36 h timepoints water (800 mL) was sampled for microbial communities, DOC, and solid phase extraction of DOM using a peristaltic pump connected to acid washed and seawater leached silicon tubing. Sample water (800 mL) was passed through a 0.22 µm Sterivex to collect bacterioplankton for downstream DNA analysis.
DOC samples were taken by collecting 35 mL of 0.22µm sterivex filtrate in acid washed, combusted, triple sample-rinsed clear glass vials. Care was made to flush each Sterivex with ~50 mL of sample water prior to collecting DOC to avoid contamination from the filter. DOC samples were then acidified with 50 µL of 4N hydrochloric acid to yield a pH of less than 3. The DOC samples were processed and analyzed via high-temperature combustion on slightly modified Shimadzu TOC-V analyzers at UCSB according to the protocol outlined in Carlson et al., 2010.
For analysis of metabolites, exactly 700 mL of the remaining 0.22 µm Sterivex filtrate was collected in acid washed 1 L polycarbonate bottles and acidified with HCl to pH < 2. A small volume (50 mL) of the acidified sample water was used to flush the lines prior to the solid phase extraction resulting in 650 mL of sample for solid phase extraction. Two bottles had less than 650 mL acidified sample water and were equalized to 500 mL solid phase extractions. The difference in volume was later corrected by the resuspension step prior to LC-MS/MS analysis. Metabolites were extracted using a 200 mg mass Bond Elut-PPL (Agilent) cartridges following Dittmar et al., 2008 and Petras et al., 2017.

Data collection

Symbiodiniaceae Quantification
Data were collected by Wesley Sparagon at University of Hawaiʻi at Mānoa.

Bacterioplankton Abundance
Data were collected by Wesley Sparagon at University of Hawaiʻi at Mānoa.

DOC
DOC data was collected by Keri Opalk of the Carlson lab at UCSB according to the protocol outlined in Carlson et al., 2010.

Microbial Community DNA Extraction, Library Prep, and Sequencing
Sample DNA extraction protocols followed those outlined in Bullington et al., 2022. For details, please see supplementary methods. Library preparation of the V4 16S rRNA gene region for amplicon sequencing was conducted at the University of Hawaiʻi at Mānoa Microbial Genomics and Analytical Laboratory using a single barcode library preparation approach with Golay barcoded forward primers and non-barcoded reverse primers. For an overview of primers and settings used, see the supplementary methods. Amplicons were pooled and sequenced using an Illumina MiSeq V3 600 paired-end cycle run at the University of Hawaiʻi at Mānoa Advanced Studies in Genomics, Proteomics and Bioinformatics facility. A total of 243 samples from this experiment as well as other experiments that occurred at the same field site and time were included in this sequencing library. All samples were amplified and sequenced in duplicate technical replicates. Method blanks had substantially lower sequence read depth (mean = 1,590 reads/sample) than samples (mean = 88,681), with samples ranging from 12,609 reads/sample to 155,685 reads/sample.

Dissolved Organic Matter Composition
DOM composition data was collected by Dr. Zach Quinlan and Irina Koester at UCSD. PPL cartridges were eluted with 2 mL methanol. Extracts were dried down with a vacuum centrifuge and redissolved with 70 µL 80% methanol:water with 1% formic acid. The two samples that had less volume were redissolved to 50 µL so that all concentrations were normalized to filtrate volume. Samples were transferred into a combusted glass insert. A 10 µL aliquot of each sample was analyzed by injection into a Vanquish ultra-high performance liquid chromatography system (UHPLC) coupled to a Q-Exactive Orbitrap Mass Spectrometer (Thermo Fisher Scientific, Bremen, Germany). Chromatographic separation was performed with a C18 core-shell column (Kinetex, 150 × 2 mm, 1.8 µm particle

	size, 100 Å pore size, Phenomenex, Torrance, USA) all using the settings and protocol described in Petras et al., 2017 and Wegley Kelly et al., 2022.
Timing and spatial scale	All data except for temperature/light, Symbiodiniaceae densities, and bacterioplankton abundance were collected at the beginning and end of the bottle incubations, which spanned a total of 36 hours from 21 May 2019 to 23 May 2019. We determined the 36 hour incubation based on previous bottle incubation experiments conducted at the field site.<br><br>Temperature and light data were collected for the flow-through aquaria pre-treatment from 14 May 2019 to 21 May 2019.<br><br>Bacterioplankton abundance data were collected throughout the 36 hour bottle incubation at 0, 2, 8, 16, 20, 24, 32, and 36 hours. These timepoints were determined based on prior bottle incubations conducted at the field site.
Data exclusions	At the final time point, two outlier samples were identified and removed from downstream 16S analysis (outliers were defined as samples whose log10 distance from the centroid of a treatment ≥ 1.5 SD above the mean log10 distance from the centroid for a given treatment).<br><br>Four total DOC samples were removed due to incorrect septa orientation which led to DOC contamination. These samples were identified by the DOC analytical lab at UCSB.
Reproducibility	Due to time constraints in the field, this experiment was not repeated. However, ongoing studies by the team have sought to identify if the broad conclusions of this study are validated in situ.
Randomization	Each experimental aquaria received all genotypes of a given coral species at a given bleaching phenotype. Aquaria were placed within their treatment (heated or ambient) water table using haphazard placement. Bottles were placed in the dark chamber during the 36 hour incubation using haphazard placement.
Blinding	Blinding was not relevant because data generated from this experiment was not obtained until long after the experiment itself was completed, and those who collected samples and data were not necessarily the same individuals who designed the experiment and analyzed the data.

Did the study involve field work? ☒ Yes ☐ No

Field work, collection and transport

Field conditions	Prior to sampling there were multiple thermal anomalies starting in December 2018 in which temperatures exceeded the thermal stress accumulation threshold level of 29 °C (Leinbach et al., 2021; Pratchett et al., 2013; Speare et al., 2021) by >1°C. Temperature trends were analyzed using the Mo'orea Coral Reef Long Term Ecological Research (MCR LTER) daily average water temperature data. Time-series data were collected from 3 sites on the MCR LTER fore-reef: FOR1, FOR4 and FOR5 (GPS location: 17°28'30.0"S 149°50'13.2"W; 17°32'49.2"S 149°46'08.4"W; 17°34'55.2"S 149°52'30.0"W; respectively). From each location, measurements from five sensors ("upper water column", "middle water column", "bottom water column", "temperature shallow", and "temperature deeper") was used to calculate the average temperature +/- one standard deviation. Bleaching was first observed in the corals adjacent to Gump Station, Mo'orea, in April 2019 (Leinbach et al., 2021) (Figure 1B). Accumulated degree heating days reached a maximum of 17°C-Days in mid-April before rapidly decreasing (Burgess et al., 2021). By the start of field collection on May 8th, 2019, the temperatures dropped below the 29 °C threshold. Corals had experienced a total of 110 days of temperatures exceeding the threshold in a period of five months (151 days).
Location	Coral nubbins from three different species (Pocillopora verrucosa, Acropora pulchra, and Porites rus) were collected in Mo'orea, French Polynesia on May 8th, 2019 immediately following a bleaching event. Pocillopora verrucosa and Acropora pulchra nubbins were collected from a common garden LTER1 site on the back-reef of Paopao Bay, Mo'orea, French Polynesia (17°28'45.0"S 149° 50'10.44"W). Porites rus nubbins were collected from the fringing reef north of Gump Station research facility (17°29'11.6"S 149° 49'31.1"W). After collection, corals were transported to the Gump Station research facility and acclimated to ambient conditions in a 1300 L flow-through water table for three days.
Access & import/export	Nubbins were collected under permits issued by the French Polynesian Government (Délégation à la Recherche) and the Haut-commissariat de la République en Polynésie Francaise (DTRT) (Protocole d'Accueil 2005-2021).
Disturbance	Corals were collected from the field using bonecutters and researchers minimized the disturbance to non-target organisms during collection.

Reporting for specific materials, systems and methods

We require information from authors about some types of materials, experimental systems and methods used in many studies. Here, indicate whether each material, system or method listed is relevant to your study. If you are not sure if a list item applies to your research, read the appropriate section before selecting a response.

Materials & experimental systems

n/a	Involved in the study	
☐	☐	Antibodies
☐	☐	Eukaryotic cell lines
☐	☐	Palaeontology and archaeology
☐	☒	Animals and other organisms
☐	☐	Clinical data
☐	☐	Dual use research of concern

Methods

n/a	Involved in the study	
☐	☐	ChIP-seq
☐	☒	Flow cytometry
☐	☐	MRI-based neuroimaging

Antibodies

Antibodies used — *Describe all antibodies used in the study; as applicable, provide supplier name, catalog number, clone name, and lot number.*

Validation — *Describe the validation of each primary antibody for the species and application, noting any validation statements on the manufacturer's website, relevant citations, antibody profiles in online databases, or data provided in the manuscript.*

Eukaryotic cell lines

Policy information about cell lines and Sex and Gender in Research

Cell line source(s) — *State the source of each cell line used and the sex of all primary cell lines and cells derived from human participants or vertebrate models.*

Authentication — *Describe the authentication procedures for each cell line used OR declare that none of the cell lines used were authenticated.*

Mycoplasma contamination — *Confirm that all cell lines tested negative for mycoplasma contamination OR describe the results of the testing for mycoplasma contamination OR declare that the cell lines were not tested for mycoplasma contamination.*

Commonly misidentified lines (See ICLAC register) — *Name any commonly misidentified cell lines used in the study and provide a rationale for their use.*

Palaeontology and Archaeology

Specimen provenance — *Provide provenance information for specimens and describe permits that were obtained for the work (including the name of the issuing authority, the date of issue, and any identifying information). Permits should encompass collection and, where applicable, export.*

Specimen deposition — *Indicate where the specimens have been deposited to permit free access by other researchers.*

Dating methods — *If new dates are provided, describe how they were obtained (e.g. collection, storage, sample pretreatment and measurement), where they were obtained (i.e. lab name), the calibration program and the protocol for quality assurance OR state that no new dates are provided.*

☐ Tick this box to confirm that the raw and calibrated dates are available in the paper or in Supplementary Information.

Ethics oversight — *Identify the organization(s) that approved or provided guidance on the study protocol, OR state that no ethical approval or guidance was required and explain why not.*

Note that full information on the approval of the study protocol must also be provided in the manuscript.

Animals and other research organisms

Policy information about studies involving animals; ARRIVE guidelines recommended for reporting animal research, and Sex and Gender in Research

Laboratory animals — *For laboratory animals, report species, strain and age OR state that the study did not involve laboratory animals.*

Wild animals — Coral nubbins from three different species (Pocillopora verrucosa, Acropora pulchra, and Porites rus) were collected using bonecutters and immedeatly transported to Gump Station research facility in coolers filled with ambient seawater. Corals were destructively sampled at the end of the experiment via flash freezing, followed by air-brushing using a water pick for Symbiodiniaceae collection.

Reporting on sex — *Indicate if findings apply to only one sex; describe whether sex was considered in study design, methods used for assigning sex. Provide data disaggregated for sex where this information has been collected in the source data as appropriate; provide overall numbers in this Reporting Summary. Please state if this information has not been collected. Report sex-based analyses where*

	performed, justify reasons for lack of sex-based analysis.
Field-collected samples	Coral nubbins from three different species (Pocillopora verrucosa, Acropora pulchra, and Porites rus) were collected using bonecutters and immedeatly transported to Gump Station research facility in coolers filled with ambient seawater. After collection, corals were transported to the Gump Station research facility and acclimated to ambient conditions in a water table for three days. Corals were destructively sampled at the end of the experiment via flash freezing, followed by air-brushing using a water pick for Symbiodiniaceae collection. Ambient temperatures and light levels were maintained throughout this period (Table S3).
Ethics oversight	Nubbins were collected under permits issued by the French Polynesian Government (Délégation à la Recherche) and the Haut-commissariat de la République en Polynésie Francaise (DTRT) (Protocole d'Accueil 2005-2021). No additional ethics oversight was necessary for work with these organisms at this research facility.

Note that full information on the approval of the study protocol must also be provided in the manuscript.

Clinical data

Policy information about clinical studies

All manuscripts should comply with the ICMJE guidelines for publication of clinical research and a completed CONSORT checklist must be included with all submissions.

Clinical trial registration	*Provide the trial registration number from ClinicalTrials.gov or an equivalent agency.*
Study protocol	*Note where the full trial protocol can be accessed OR if not available, explain why.*
Data collection	*Describe the settings and locales of data collection, noting the time periods of recruitment and data collection.*
Outcomes	*Describe how you pre-defined primary and secondary outcome measures and how you assessed these measures.*

Dual use research of concern

Policy information about dual use research of concern

Hazards

Could the accidental, deliberate or reckless misuse of agents or technologies generated in the work, or the application of information presented in the manuscript, pose a threat to:

No	Yes	
☒	☐	Public health
☒	☐	National security
☒	☐	Crops and/or livestock
☒	☐	Ecosystems
☒	☐	Any other significant area

Experiments of concern

Does the work involve any of these experiments of concern:

No	Yes	
☒	☐	Demonstrate how to render a vaccine ineffective
☒	☐	Confer resistance to therapeutically useful antibiotics or antiviral agents
☒	☐	Enhance the virulence of a pathogen or render a nonpathogen virulent
☒	☐	Increase transmissibility of a pathogen
☒	☐	Alter the host range of a pathogen
☒	☐	Enable evasion of diagnostic/detection modalities
☒	☐	Enable the weaponization of a biological agent or toxin
☒	☐	Any other potentially harmful combination of experiments and agents

ChIP-seq

Data deposition

☐ Confirm that both raw and final processed data have been deposited in a public database such as GEO.

☐ Confirm that you have deposited or provided access to graph files (e.g. BED files) for the called peaks.

Data access links	*For "Initial submission" or "Revised version" documents, provide reviewer access links. For your "Final submission" document,*

Data access links	*provide a link to the deposited data.*
Files in database submission	*Provide a list of all files available in the database submission.*
Genome browser session (e.g. UCSC)	*Provide a link to an anonymized genome browser session for "Initial submission" and "Revised version" documents only, to enable peer review. Write "no longer applicable" for "Final submission" documents.*

Methodology

Replicates	*Describe the experimental replicates, specifying number, type and replicate agreement.*
Sequencing depth	*Describe the sequencing depth for each experiment, providing the total number of reads, uniquely mapped reads, length of reads and whether they were paired- or single-end.*
Antibodies	*Describe the antibodies used for the ChIP-seq experiments; as applicable, provide supplier name, catalog number, clone name, and lot number.*
Peak calling parameters	*Specify the command line program and parameters used for read mapping and peak calling, including the ChIP, control and index files used.*
Data quality	*Describe the methods used to ensure data quality in full detail, including how many peaks are at FDR 5% and above 5-fold enrichment.*
Software	*Describe the software used to collect and analyze the ChIP-seq data. For custom code that has been deposited into a community repository, provide accession details.*

Flow Cytometry

Plots

Confirm that:

- [x] The axis labels state the marker and fluorochrome used (e.g. CD4-FITC).
- [x] The axis scales are clearly visible. Include numbers along axes only for bottom left plot of group (a 'group' is an analysis of identical markers).
- [x] All plots are contour plots with outliers or pseudocolor plots.
- [x] A numerical value for number of cells or percentage (with statistics) is provided.

Methodology

Sample preparation

Symbiodiniaceae Quantification
Following exudation corals were removed from the aquaria and airbrushed using 0.22uM filtered seawater to collect tissue slurry for downstream Symbiodiniaceae quantification. Samples were frozen at -40 °C for 14 days prior to transportation to University of Hawaiʻi at Mānoa campus where they were frozen at -80 °C for 23 months prior to flow cytometry processing. coral nubbins were flash-frozen and airbrushed using 0.22 µm filtered seawater. Tissue slurries were analyzed using flow cytometry following the protocol outlined in Fox et al., 2021. In brief, samples were thawed, briefly homogenized using a vortex machine, and 200 µL of each sample being aliquoted into 96-well round-bottom flow cytometry plates. Symbiodiniaceae slurries were run on a Beckman Coulter CytoFLEX S Flow Cytometer (Beckman Coulter, Product No: B78560). The Chlorophyll-a emissions PMT and the two scatter detectors for the violet laser were used in conjunction to count Symbiodiniaceae cells, delineated as distinct populations of large cells with high specific chlorophyll a content. Voltages and gating were manually determined to enable easy identification of this population using a FSC gain of 100, a SSC gain of 100, and a Chla gain of 50. Chla values had a lower threshold of 20,000. Density plots of Chla vs. FSC were gated on the easily distinguishable population of SYBR green stained bacteria (Figure S1b).

Bacterioplankton Quantification
1 mL of each seawater sample was fixed with 16 µL of 32% paraformaldehyde PFA. Samples for bacterioplankton abundance were flash-frozen at -40 °C for 14 days prior to transportation to University of Hawaiʻi at Mānoa campus where they were frozen at -80 °C for six months prior to flow cytometry processing. Fixed microbial abundance samples were thawed, and 200 µL of each sample aliquoted and stained with 2 µL 100X SYBR Green. The BL1 detector for the blue laser (488 nm) and the SSC detector for the violet laser (405 nm) were used in conjunction to elucidate bacterial abundances. Voltages and gating were manually determined to enable easy identification of SYBR green stained bacterioplankton populations using a BL1 voltage of 2,625 mV and a SSC voltage of 2,500 mV. Density plots of BL1 vs. SSC were gated on the easily distinguishable population of SYBR green stained bacteria (Figure S1a).

Instrument

Symbiodiniaceae Quantification
Beckman Coulter CytoFLEX S Flow Cytometer (Beckman Coulter, Product No: B78560)

Bacterioplankton Quantification
Attune Acoustic Focusing Cytometer (Applied Biosystems, Part No. 4445280ASR)

Software

CytExpert Software was used for Symbiodiniaceae data collection and Attune Cytometric Software v2.1 was used for bacterioplankton data collection.

Cell population abundance | Sorting was not conducted.

Gating strategy | Bacterioplankton Quantification
The BL1 detector for the blue laser (488 nm) and the SSC detector for the violet laser (405 nm) were used in conjunction to elucidate bacterial abundances. Voltages and gating were manually determined to enable easy identification of SYBR green stained bacterioplankton populations using a BL1 voltage of 2,625 mV and a SSC voltage of 2,500 mV. Density plots of BL1 vs. SSC were gated on the easily distinguishable population of SYBR green stained bacteria (Figure S1a).

Symbiodiniaceae Quantification
The Chlorophyll-a emissions PMT and the two scatter detectors for the violet laser were used in conjunction to count Symbiodiniaceae cells, delineated as distinct populations of large cells with high specific chlorophyll a content. Voltages and gating were manually determined to enable easy identification of this population using a FSC gain of 100, a SSC gain of 100, and a Chla gain of 50. Chla values had a lower threshold of 20,000. Density plots of Chla vs. FSC were gated on the easily distinguishable population of SYBR green stained bacteria (Figure S1b).

☒ Tick this box to confirm that a figure exemplifying the gating strategy is provided in the Supplementary Information.

Magnetic resonance imaging

Experimental design

Design type | *Indicate task or resting state; event-related or block design.*

Design specifications | *Specify the number of blocks, trials or experimental units per session and/or subject, and specify the length of each trial or block (if trials are blocked) and interval between trials.*

Behavioral performance measures | *State number and/or type of variables recorded (e.g. correct button press, response time) and what statistics were used to establish that the subjects were performing the task as expected (e.g. mean, range, and/or standard deviation across subjects).*

Acquisition

Imaging type(s) | *Specify: functional, structural, diffusion, perfusion.*

Field strength | *Specify in Tesla*

Sequence & imaging parameters | *Specify the pulse sequence type (gradient echo, spin echo, etc.), imaging type (EPI, spiral, etc.), field of view, matrix size, slice thickness, orientation and TE/TR/flip angle.*

Area of acquisition | *State whether a whole brain scan was used OR define the area of acquisition, describing how the region was determined.*

Diffusion MRI ☐ Used ☐ Not used

Preprocessing

Preprocessing software | *Provide detail on software version and revision number and on specific parameters (model/functions, brain extraction, segmentation, smoothing kernel size, etc.).*

Normalization | *If data were normalized/standardized, describe the approach(es): specify linear or non-linear and define image types used for transformation OR indicate that data were not normalized and explain rationale for lack of normalization.*

Normalization template | *Describe the template used for normalization/transformation, specifying subject space or group standardized space (e.g. original Talairach, MNI305, ICBM152) OR indicate that the data were not normalized.*

Noise and artifact removal | *Describe your procedure(s) for artifact and structured noise removal, specifying motion parameters, tissue signals and physiological signals (heart rate, respiration).*

Volume censoring | *Define your software and/or method and criteria for volume censoring, and state the extent of such censoring.*

Statistical modeling & inference

Model type and settings | *Specify type (mass univariate, multivariate, RSA, predictive, etc.) and describe essential details of the model at the first and second levels (e.g. fixed, random or mixed effects; drift or auto-correlation).*

Effect(s) tested | *Define precise effect in terms of the task or stimulus conditions instead of psychological concepts and indicate whether ANOVA or factorial designs were used.*

Specify type of analysis: ☐ Whole brain ☐ ROI-based ☐ Both

Statistic type for inference (See Eklund et al. 2016) | *Specify voxel-wise or cluster-wise and report all relevant parameters for cluster-wise methods.*

Correction

Describe the type of correction and how it is obtained for multiple comparisons (e.g. FWE, FDR, permutation or Monte Carlo).

Models & analysis

n/a	Involved in the study
☐	☐ Functional and/or effective connectivity
☐	☐ Graph analysis
☐	☐ Multivariate modeling or predictive analysis

Functional and/or effective connectivity

Report the measures of dependence used and the model details (e.g. Pearson correlation, partial correlation, mutual information).

Graph analysis

Report the dependent variable and connectivity measure, specifying weighted graph or binarized graph, subject- or group-level, and the global and/or node summaries used (e.g. clustering coefficient, efficiency, etc.).

Multivariate modeling and predictive analysis

Specify independent variables, features extraction and dimension reduction, model, training and evaluation metrics.
